# Targeting CRL4 suppresses chemoresistant ovarian cancer growth by inducing mitophagy

**DOI:** 10.1038/s41392-022-01253-y

**Published:** 2022-12-09

**Authors:** Yang Meng, Lei Qiu, Xinyi Zeng, Xiaoyan Hu, Yaguang Zhang, Xiaowen Wan, Xiaobing Mao, Jian Wu, Yongfeng Xu, Qunli Xiong, Zhixin Chen, Bo Zhang, Junhong Han

**Affiliations:** 1 Research Laboratory of Tumor Epigenetics and Genomics, Department of General Surgery, Frontiers Science Center for Disease-related Molecular Network and National Clinical Research Center for Geriatrics, State Key Laboratory of Biotherapy and Cancer Center, West China Hospital, Sichuan University, Chengdu, 610041 China; 2 Division of Cancer Cell Biology, The Graduate School of Frontier Sciences, The University of Tokyo, 4-6-1 Shirokanedai, Minato-ku, Tokyo, 108-8639 Japan; 3 Division of Hematology/Oncology, Department of Medicine, Virginia Commonwealth University, Richmond, VA USA; 4 Abdominal Oncology Ward, Cancer Center, West China Hospital of Sichuan University, Chengdu, 610041 China

**Keywords:** Gynaecological cancer, Cancer therapy, Cell biology

## Abstract

Chemoresistance has long been the bottleneck of ovarian cancer (OC) prognosis. It has been shown that mitochondria play a crucial role in cell response to chemotherapy and that dysregulated mitochondrial dynamics is intricately linked with diseases like OC, but the underlying mechanisms remain equivocal. Here, we demonstrate a new mechanism where CRL4^CUL4A/DDB1^ manipulates OC cell chemoresistance by regulating mitochondrial dynamics and mitophagy. CRL4^CUL4A/DDB1^ depletion enhanced mitochondrial fission by upregulating AMPKα^Thr172^ and MFF^Ser172/Ser146^ phosphorylation, which in turn recruited DRP1 to mitochondria. CRL4^CUL4A/DDB1^ loss stimulated mitophagy through the Parkin-PINK1 pathway to degrade the dysfunctional and fragmented mitochondria. Importantly, CRL4^CUL4A/DDB1^ loss inhibited OC cell proliferation, whereas inhibiting autophagy partially reversed this disruption. Our findings provide novel insight into the multifaceted function of the CRL4 E3 ubiquitin ligase complex in regulating mitochondrial fission, mitophagy, and OC chemoresistance. Disruption of CRL4^CUL4A/DDB1^ and mitophagy may be a promising therapeutic strategy to overcome chemoresistance in OC.

## Introduction

Ovarian cancer (OC) is the 11th most common cancer in women and the fifth leading cause of cancer-related death in the world,^1^ with an estimation of 314,000 newly diagnosed cases and approximately 207,000 deaths in 2020.^2^ OC is the most fatal gynecologic cancer. Owing to its asymptomatic development, such as equivocal signs and symptoms that can “masquerade” as other nonmalignant conditions and lack of an explicit screening tool, the majority of OC patients are diagnosed in advanced stages, even in the developed world.^3^ Cisplatin is still used as the frontline treatment for OC. However, chemoresistance often occurs, resulting in a five-year survival of only 20–30% without significant improvement over the years in patients with advanced OC.^4–6^

Mitochondria act as cellular powerhouses and signaling organelles and thus play important roles in effective cell bioenergetics, reduction-oxidation balance, calcium homeostasis, and programmed cell death.^7^ Previous discoveries paved the way for the idea that dysregulation of mitochondria played a role in cancer progression and chemoresistance, as now corroborated by ample evidence.^8,9^ Mitochondrial networks are dynamically remodeled through the opposing processes of fusion and fission in response to the microenvironment.^10^ Recently, multiple mitochondrial quality control mechanisms have been discovered, including Parkin-dependent and Parkin-independent mechanisms, mitochondrial fission and fusion, mitochondria-derived vesicles, and mitochondrial spheroid formation.^11^ Mitophagy, a well-studied type of cargo-specific autophagy, acts as the important mitochondrial quality control mechanism that removes damaged mitochondria.^12^ The Parkin-dependent mitophagy pathway, which requires both Parkin and Pink1, has been shown to maintain mitochondrial homeostasis.^11^ Additionally, recent studies have uncovered several key molecules involved in the fission and fusion of mitochondria. Mitochondrial outer membrane fusion is driven by Mitofusins 1 and 2 (Mfn1 and Mfn2, respectively), which are crucial molecules for tethering the outer membrane to the endoplasmic reticulum.^13,14^ The GTPase dynamin-related protein 1 (DRP1) is often considered the master regulator of mitochondrial fission and contributes to the mitochondrial network.^15^ DRP1 phosphorylation plays a crucial role in DRP1 activation, which is required for the recruitment of DRP1 to mitochondria. Research studies have revealed that DRP1 phosphorylation at Ser616 activates DRP1, promoting DRP1 translocation from the cytosol to the mitochondrial outer membrane and inducing mitochondrial fission.^16,17^ Conversely, DRP1 phosphorylation at Ser637 inactivates DRP1 and suppresses mitochondrial fission.^18^ Phosphoglycerate mutase family member 5,^19^ AMPK,^20–22^ MAPK,^23,24^ and cyclin-dependent kinase 1/cyclin B1 (Cdk1/cyclin B1)^17^ are several key molecules involved in DRP1 phosphorylation during mitochondrial fission. Reversible phosphorylation of DRP1 may play a crucial role in mediating its subcellular localization and mitochondrial fission, but many aspects of mitochondrial fission required for DRP1 in cancer chemoresistance are still promiscuous.

Cullin-RING ubiquitin ligases are a family of E3 ubiquitin ligase complexes that are responsible for degrading 20% of the proteins in the cell.^25^ CRL4 is a complex composed of a cullin scaffold protein Cullin-4A/B (CUL4A/B), attached to a homeobox-leucine zipper protein (ROC1) at its C-terminus, whereas its N-terminus is associated with DNA damage-binding protein 1 (DDB1), as well as an adapter protein DCAF, which recruits a large family of substrate receptors.^26^ CRL4^CUL4A/DDB1^, a well-defined E3 ubiquitin ligase, is primarily involved in DNA damage repair of the cell, DNA replication, chromatin remodeling, and the biological processes involved in maintaining genomic stability.^26–28^ Recently, accumulated data suggest that CRL4^CUL4A/DDB1^ may be involved in the regulation of mitochondrial structure and functions. CRL activation requires Cullin neddylation in eukaryotes. Protein neddylation is an important posttranslational modification catalyzed by an E1 NEDD8-activating enzyme (NAE), an E2 NEDD8-conjugating enzyme, and an E3 NEDD8 ligase.^26^ Intriguingly, emerging evidence indicates that mitochondrial homeostasis is subjected to supervision by neddylation and CRLs.^29–32^ Therefore, targeting neddylation pathways to inactivate CRLs has gradually become an attractive anticancer regimen.^33^ MLN4924, an NAE inhibitor, directly binds to NAE to form a covalent NEDD8-MLN4924 adduct and efficiently inhibits the Cullin neddylation modification.^25^ Pan et al. demonstrated that inhibition of CRL4^CDT2^ by MLN4924 might be used as an effective anticancer strategy for targeted OC therapy.^34^ Additionally, our previous work confirmed that CRL4 mediated OC chemoresistance by targeting the antiapoptotic protein BIRC3.^35^ Nevertheless, it remains unknown whether CRL4 and its neddylation are involved in regulating mitochondrial function and chemoresistant OC.

In this study, we uncovered a novel regulatory mechanism by which CRL4^CUL4A/DDB1^ E3 ligase influenced OC chemotherapy resistance. We found that CRL4^CUL4A/DDB1^ was significantly upregulated in cisplatin-resistant OC cells (OCCs) and that knockdown of CRL4^CUL4A/DDB1^ inhibited cell proliferation, disrupted mitochondrial morphology and functions, and induced mitophagy in cisplatin-resistant OCCs. Interestingly, we found that the knockdown of CRL4^CUL4A/DDB1^ led to increase expression of the mitochondrial fission protein DRP1 and decrease phosphorylation of DRP1^Ser637^, which correlated with increased mitochondrial fission via the AMPK-MFF-DRP1 signaling axis. This implies a mechanistic link between CRL4^CUL4A/DDB1^ and the Parkin/PINK1 signaling pathway in mediating mitophagy in OCCs.

## Results

### CRL4^CUL4A/DDB1^ is correlated with worse OC outcome and is critical for its cisplatin resistance

**Fig. 1:**
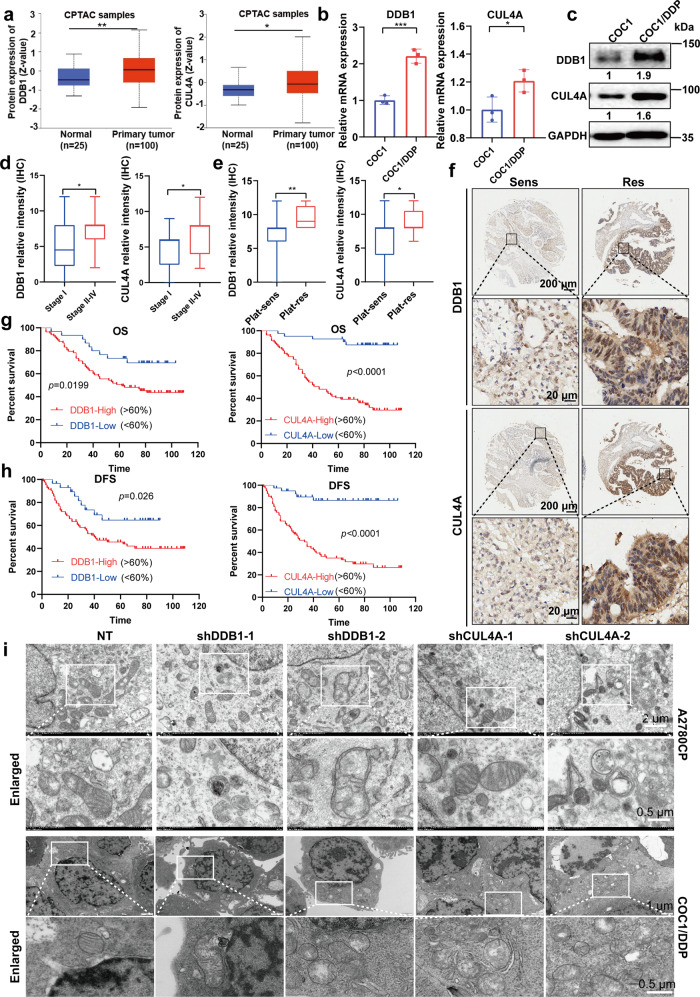
CRL4^CUL4A/DDB^1 is critical for cisplatin resistance and mitochondrial morphology maintenance of OCCs. **a** Protein expression of DDB1 and CUL4A in normal ovaries and OC tissue samples based on the CPTAC dataset. Raw data were obtained from the OncoLnc database. *p < 0.01, **p < 0.01. The significance of expression level difference was determined using *a t*-test. **b** Relative mRNA expression of DDB1 and CUL4A detected by qPCR in COC1/DDP and COC1 cell lines. Data represent mean ± SEM normalized to 18S. *p < 0.01, **p < 0.01, ***p < 0.001. The significance of expression level difference was determined using a *t*-test. **c** Relative DDB1 and CUL4A protein expression levels in COC1/DDP and COC1 cell lines detected by Western Blot. GAPDH was used as a loading control. **d**, **e** Box-and-whiskers plot showing a relationship between (left panel) DDB1 and (right panel) CUL4A expression and **d** serous tumor stage and **e** platinum (Plat) resistance. *n* = 113 for DDB1 staining (*n* = 8 for platinum-resistant, *n* = 105 for platinum-sensitive) *n* = 120 for CUL4A staining (*n* = 9 for platinum-resistant, *n* = 111 for platinum-sensitive). **f** Immunohistochemical staining analysis of (upper panel) DDB1 and (lower panel) CUL4A expression in tumor cells in Plat-sensitive (Sens) and Plat-resistant (Res) serous tumors. *n* = 113 for DDB1 staining (*n* = 8 for platinum-resistant, *n* = 105 for platinum-sensitive) *n* = 120 for CUL4A staining (*n* = 9 for platinum-resistant, *n* = 111 for platinum-sensitive). **g** Overall survival (OS) and **h** Diseases free survival (DFS) in patients with serous ovarian cancer expressing high (>60% positive cells) or low (<60% positive cells) levels of (left) DDB1 or (right) CUL4A. *n* = 113 for DDB1 staining (*n* = 8 for platinum-resistant, *n* = 105 for platinum-sensitive) *n* = 120 for CUL4A staining (*n* = 9 for platinum-resistant, *n* = 111 for platinum-sensitive). **i** Representative images showing mitochondrial morphology in (upper panel) A2780CP and (lower panel) COC1/DDP cells with CRL4-knockdown by transmission electron microscope assay

Our previous study demonstrated that CRL4 mediated the apoptosis of cisplatin-resistant OC by targeting the protein BIRC3.^35^ To further determine the role of CRL4 in OC, we analyzed CUL4A and DDB1 expression in OC and matched normal tissue samples using the Oncomine database. The results showed that transcription of CUL4A and DDB1 was significantly higher in tumor tissue than in normal tissue (Supplementary Fig. 1a, b). We verified CUL4A and DDB1 expression by analyzing data from the TCGA database. Data in the CPTAC dataset indicated that CUL4A (*p < 0.05) and DDB1 (**p < 0.01) were highly expressed in OC compared with normal ovarian tissue (Fig. 1a). Further tumor grade analyses of multiple clinicopathological features of TCGA-OV samples in the UALCAN database showed that CUL4A and DDB1 expression increased in tumors compared to paracancerous tissue in a non-grade-dependent manner (Supplementary Fig. 1c, d). To determine the importance of CRL4^CUL4A/DDB1^ in OC prognosis, we first analyzed data from the Kaplan–Meier plotter website^36^ (http://kmplot.com/). We classified OC cases into high DDB1/CUL4A and low DDB1/CUL4A expression groups and investigated the correlation of CRL4^CUL4A/DDB1^ expression with the prognosis of OC patients. As shown in Supplementary Fig. 1e, high CUL4A expression correlated with worse overall survival (OS) and progression-free survival (PFS) for OC patients, whereas DDB1 did not show a significant correlation with either OS or PFS in this dataset.

To further determine the role of CRL4^CUL4A/DDB1^ in OC cisplatin resistance, we verified CUL4A and DDB1 expression in OC cells and in OC tissue microarray samples. Consistent with our previous study, where we demonstrated higher CUL4A and DDB1 expression in cisplatin-resistant A2780CP cells compared to cisplatin-sensitive A2780 cells, we observed a significant increase in CUL4A and DDB1 expression in cisplatin-resistant COC1/DDP cells compared to the cisplatin-sensitive COC1 cells (Fig. 1b, c). We also validated CRL4^CUL4A/DDB1^ protein expression in OC clinical samples collected on a tissue microarray with immunohistochemical (IHC) staining. In agreement with our analyses with online datasets, Stage I serous tumors displayed lower DDB1 and CUL4A expression, whereas late-stage serous tumors (Stages II-IV) showed significantly higher DDB1 and CUL4A expression (Fig. 1d). Moreover, higher CRL4^CUL4A/DDB1^ nuclear staining was also observed in platinum-resistant patient tumor samples (Fig. 1e, f) and was correlated with reduced overall and disease-free survival (DFS) (Fig. 1g, h). When comparing each of the OC histotypes to the samples with mixed histotypes by Mann–Whitney Test, we observed a significantly higher CUL4A expression in serous adenocarcinoma (*p* = 0.01) and a lower DDB1 expression in mucinous adenocarcinoma (*p* = 0.004), whereas no significant differences in CUL4A or DDB1 expression was observed in other histotypes (Supplementary Fig. 1g, h). Among the OC tissues, the proportion of serous adenocarcinoma was over 50%, consistent with its high proportion among OC cases. It is also the most malignant histotype among OC histotypes, suggesting that CRL4^CUL4A/DDB1^ is indeed important for OC prognosis. These results further support our conclusion in the previous study that CRL4^CUL4A/DDB1^ plays a critical role in promoting OC progression and in OC cisplatin resistance.^35^

To further confirm CRL4 contribution in OC cisplatin treatment, we used two cisplatin-resistant OC cell lines (A2780CP and COC1/DDP) along with their corresponding parental cisplatin-sensitive OC cell lines (A2780 and COC1) and examined their IC_50_ in response to cisplatin treatment for 24 h. Our results confirmed that A2780CP and COC1-DDP cells were more resistant to cisplatin treatment than A2780 and COC1 cells, respectively (Supplementary Fig. 2a). Cullin4 E3 ubiquitin ligase has been reported to be upregulated in OC and is proposed as a potential drug target in OC.^34,35^ Our previous results showed that CRL4 knockdown was able to reverse the cisplatin resistance of A2780CP cells.^35^ To further validate our conclusion, we knocked down CRL4^CUL4A/DDB1^ in the four cell lines mentioned above (Supplementary Fig. 2b–g) and found that CUL4A/DDB1 loss significantly affected EdU incorporation in cells (Supplementary Fig. 2h–m). Indeed, the knockdown of either CUL4A or DDB1 in cisplatin-resistant A2780CP and COC1/DDP cells impaired cell viability in response to cisplatin treatment (Supplementary Fig. 2n, o). Interrupting protein neddylation with MLN4924 has been reported as a novel strategy to target cisplatin resistance in OC. MLN4924, also known as pevonedistat, directly inhibits neddylation and indirectly inhibits CRLs.^25^ Our results revealed that MLN4924 treatment enhanced the sensitivity of cisplatin-resistant A2780CP and COC1/DDP cells to cisplatin treatment (Supplementary Fig. 2p). Of interest, transmission electron microscopy showed that knockdown of CRL4^CUL4A/DDB1^ induced mitochondrial fragmentation or swelling (Fig. 1i). These results suggest that CRL4^CUL4A/DDB1^ may be involved in the regulation of mitochondrial homeostasis in cisplatin-resistant OCCs.

### Knockdown of CRL4^CUL4A/DDB1^ significantly impaired mitochondrial function in cisplatin-resistant OCCs

**Fig. 2:**
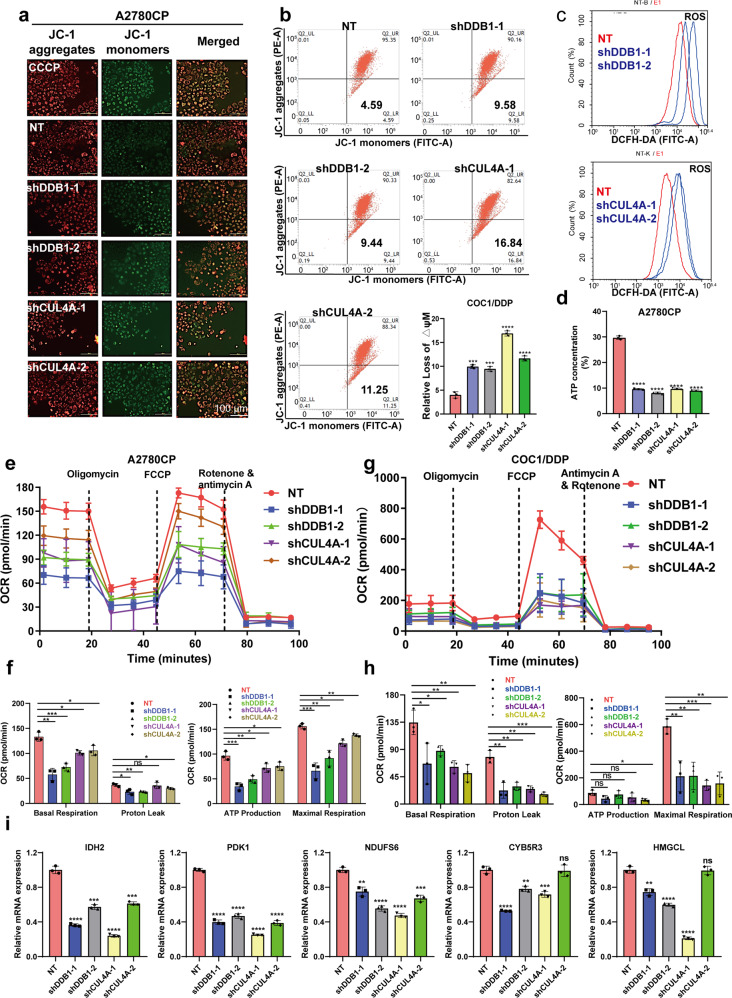
Knocking down CRL4^CUL4A/DDB1^ significantly impaired the mitochondrial function of cisplatin-resistant OCCs. **a** Mitochondrial membrane potential in A2780CP cells determined with JC-1 probe after CRL4^CUL4A/DDB1^ knockdown. CCCP (Carbonyl cyanide 3-chlorophenylhydrazone) was used as a positive control. Normal mitochondrial membrane potential (MMP) is shown in red with JC-1 dimers (JC-1 aggregates) and depolarized membrane potential is shown in green in JC-1 monomers, where a fluorescent color change from red to green indicates a decrease of MMP. Scale Bar: 20 μm. **b** Mitochondrial membrane potential detected by flow cytometric analysis in intact cells treated as in (**a**) and the quantification. Normal mitochondrial membrane potential (MMP) is shown in JC-1 dimers (JC-1 aggregates) and depolarized membrane potential is shown in JC-1 monomers. Data represent mean ± SEM with three replicates, **p < 0.01, ***p < 0.001, ****p < 0.0001. **c** The ROS accumulation levels detected by DCAF probe using flow cytometry in A2780CP cells lacking CRL4^CUL4A/DDB1^. **d** The ATP level detected by multifunctional imaging enzyme labeling in A2780CP cells knocking down CRL4^CUL4A/DDB1^. **e**, **g** A representative graph of the oxygen consumption rate (OCR) throughout the mitochondrial-stress test using Seahorse XF24 analyzer in **e** A2780CP and **g** COC1/DDP CRL4-knockdown cells. Mitochondrial inhibitors were added to the media to assess respiratory parameters with indicated time. **f**, **h** Oxygen consumption rates (OCR) of basal respiration, proton leak, ATP production, and mitochondrial maximal respiration quantified from the mitochondrial-stress test are shown **f** A2780CP and **h** COC1/DDP CRL4-knockdown cells. Data were presented as the mean ± SEM Group differences are analyzed by the two-tailed Student’s *t*-test. **i** Transcriptional levels of TCA- and oxidative phosphorylation-associated enzymes based on RNA-seq data analysis

Several previous studies have shown that CRL4B regulates mitochondrial function.^37,38^ Reactive oxygen species (ROS) overproduction is a phenotypic change in cells treated with the antitumor agent MLN4924. Mechanistic analyses revealed the antagonistic effect of antioxidants on this excess apoptosis, suggesting that ROS overproduction, especially in mitochondria, was the principal cause of the augmentation.^39^ To ascertain the mitochondrial alterations in response to CRL4 depletion in OCCs, we measured mitochondrial membrane potential (MMP) and ROS generation to address functional alterations. We found that mitochondria in CRL4^CUL4A/DDB1^ knockdown cells produced more superoxide than those cells transfected with nontarget (NT) shRNA (Fig. 2c). Compared to the NT control group, CRL4^CUL4A/DDB1^ knockdown cells showed a decline in MMP as determined by the uptake of JC-1 (Fig. 2a, b). MLN4924 treatment induced the same change pattern of ROS and MMP in OCCs (Supplementary Fig. 3a, b). Since mitochondria act as energy factories, we also examined the ATP level of mitochondria in CRL4^CUL4A/DDB1^ knockdown cells. Consistent with MMP reduction, mitochondrial function was aberrant in CRL4^CUL4A/DDB1^ knockdown cells, as evidenced by decreased ATP levels (Fig. 2d). Supporting these results, CRL4^CUL4A/DDB1^ knockdown significantly diminished the baseline oxygen consumption rate (OCR), proton leak, ATP production, and maximal respiration compared to the NT control group in the cisplatin-resistant A2780CP and COC1/DDP cell lines (Fig. 2e–h). Of note, CRL4 knockdown significantly reduced transcriptional levels of metabolic enzymes in the Krebs cycle or oxidative phosphorylation process, including IDH2, PDK1, and NDUFS6 (Fig. 2i). Additionally, both mitochondrial enzymes CYB5R3 and HMGCL, had similar changes (Fig. 2i). Despite the significant association of CUL4A or DDB1 depletion with mitochondrial dysfunction in OCCs, we did not observe obvious mitochondrial alterations in CUL4A- or DDB1-overexpressing A2780 cells (Supplementary Fig. 3c–g). Further experiments, such as seahorse oxygen consumption rates assay, are needed to validate its oxidative phosphorylation ability. These results suggest that dysregulation of CRL4 E3 ligases functionally impairs the mitochondrial respiratory capacity of cisplatin-resistant OCCs.

### Knockdown of CRL4^CUL4A/DDB1^ induced mitochondrial fragmentation

**Fig. 3:**
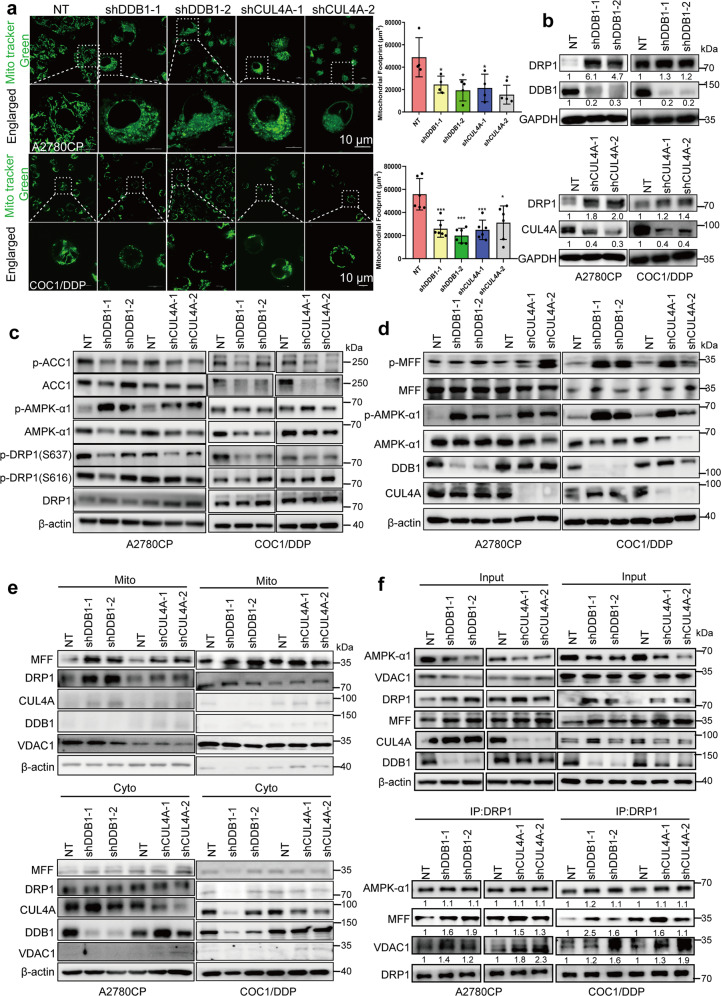
Knocking down CRL4^CUL4A/DDB1^ leads to mitochondrial fragmentation in cisplatin-resistant OCCs. **a** (Left panel) Representative images of mitochondrial morphology in (upper panel) A2780CP and (lower panel) COC1/DDP cells with CRL4^CUL4A/DDB1^ knockdown stained with MitoTracker Green. Images were captured by a laser confocal microscope. Results represent the mean from three independent experiments, measured in quadruplicate. (Right panel) Quantification analyses of the mitochondrial footprint in CRL4-knockdown (upper panel) A2780CP and (lower panel) COC1/DDP cells. The significance of differences was calculated using Student’s *t*-test (***p < 0.001). **b** DRP1 expression level in CRL4-knockdown cells detected by western blot, GAPDH serves as an internal loading control. Protein levels were quantified with Image J software and normalized to the control group (NT). **c** Immunoblot detection of ACC1, p-ACC1, CUL4A, DDB1, DRP1, p-DRP1^Ser616^, p-DRP1^Ser637^, AMPK-α1, and p-AMPK-α1 in (left panel) A2780CP and (right panel) COC1/DDP cells with CRL4^CUL4A/DDB1^ knockdown. β-actin serves as an internal loading control. **d** Immunoblot detection of MFF, p-MFF, CUL4A, DDB1, AMPK-α1, and p-AMPK-α1 in (left panel) A2780CP and (right panel) COC1/DDP cells with CRL4^CUL4A/DDB1^ knockdown. **e** Immunoblotting analysis of DRP1 and MFF in the cytosolic (Cyto) and mitochondrial (Mito) fractions of A2780CP cells with CRL4^CUL4A/DDB1^ knockdown. **f** Immunoblot detection of DRP1, AMPK-α1, and VDAC1 in (upper panel) input or (lower panel) immunoprecipitated samples in the NT control or CRL4^CUL4A/DDB1^ knockdown (left panel) A2780CP and (right panel) COC1/DDP cells. β-actin serves as a loading control

Mitochondria are dynamic organelles that continuously fuse and fragment during cell life, appearing in situ as short round-shaped, or elongated organelles with a major axis that can reach 5 μm.^40^ Many functions of mitochondria are intimately linked to their morphology. Qian and colleagues showed that platinum-based therapy induced an increase in mitochondrial fragmentation in chemosensitive OCCs but not in chemoresistant OCCs.^41^ Therefore, we evaluated the morphology of mitochondria in cisplatin-resistant OCC lines. Compared with the normal tubular mitochondria in the control group, CRL4^CUL4A/DDB1^ depletion led to an increased number of mitochondria with ring-shaped structures (Figs. 1i, 3a), suggesting the occurrence of mitochondrial fission or even fragmentation. Likewise, the mitochondrial footprints of OCC lines significantly decreased in response to MLN4924 treatment (Supplementary Fig. 4a–c). Plenty of evidence showed that the molecular machinery regulating mitochondrial dynamics comprised large dynamin-like guanosine triphosphatases (GTPases) mediating the fusion and fission of both the outer mitochondrial membrane and the inner membrane.^42^ Next, we investigated the mechanism by which dynamin-related protein 1 (DRP1) changed mitochondrial morphology. Our results revealed that knockdown of CRL4^CUL4A/DDB1^ or MLN4924 treatment significantly augmented DRP1 expression level in cisplatin-resistant OCC lines (Fig. 3b and Supplementary Fig. 4d, respectively). Previous studies have shown that cisplatin or paclitaxel causes OC cell death by reinforcing mitochondrial fragmentation and reducing DRP1 phosphorylation at serine 637 (p-DRP1^Ser637^).^43,44^ Thus, we examined the level of p-DRP1^Ser616/Ser637^ in OCCs with CRL4^CUL4A/DDB1^ knockdown. We found that p-DRP1^Ser637^ was markedly diminished in CRL4^CUL4A/DDB1^ knockdown cisplatin-resistant OCCs (Fig. 3c). Nevertheless, the level of p-DRP1^Ser616^ barely changed in response to CRL4^CUL4A/DDB1^ knockdown (Fig. 3c).

Some stimuli that activate AMP-activated protein kinase (AMPK) can also induce mitochondrial fission, suggesting a potential link between AMPK and mitochondrial fission.^45^ We found that the knockdown of CRL4 dramatically disrupted the expression level of AMPK-α1 but enhanced the level of p-AMPKα^Thr172^. It has been reported that MFF (mitochondrial fission factor) is phosphorylated at Ser172 sites by activated-AMPK, resulting in DRP1 translocation from cytosol to the mitochondria and promoting the process of mitochondrial fission.^46,47^ Thus, we then detected the level of MFF and p-MFF by western blotting in A2780CP and COC1/DDP cell lines. As shown in Fig. 3d, the knockdown of CRL4^CUL4A/DDB1^ significantly augmented the phosphorylation of AMPK and MFF. To further determine the mechanism underlying CRL4-knockdown-induced mitochondrial fission, we used a specific and potent AMPK activator, GSK-621, that can markedly increase phosphorylation at AMPKα^Thr172^.^48^ As expected, GSK-621 treatment for 24 h markedly increased the level of p-AMPKα^Thr172^ and p-MFF^Ser172/146^ in A2780CP and COC1/DDP cell lines (Supplementary Fig. 4e). Additionally, GSK-621 treatment decreased the level of p-DRP1^Ser637^ and hardly affected DRP1 expression in A2780CP and COC1/DDP cells. Congruent with our previous study, cisplatin treatment promoted the level of CRL4^CUL4A/DDB1^ expression, and slightly affected the expression of AMPK, p-AMPKα^Thr172^, MFF, p-MFF^Ser172/146^, DRP1, and p-DRP1^Ser637^ (Supplementary Fig. 4e). Consistent with these observations, we examined the subcellular localization of MFF and DRP1 in CRL4^CUL4A/DDB1^-depleted OCCs and found that knockdown of CRL4^CUL4A/DDB1^ increased MFF and DRP1 mitochondrial localization (Fig. 3e and Supplementary Fig. 4f). It has been shown that DRP1 can be acetylated at K642, which in turn activates DRP1 through phosphorylation, its translocation to mitochondria, and its oligomerization.^49^ Constitutively activated DRP1 showed higher GTPase activity and induced mitochondrial fission by binding with VDAC1 on mitochondria.^49^ Thus, we evaluated the interaction between DRP1 and the mitochondrial outer membrane protein VDAC1, which is known to regulate apoptosis. Co-Immunoprecipitation results showed that CRL4 depletion enhanced the interaction among DRP1, MFF, and VDAC1 in OCCs, and we also observed a slight increase in the interaction between DRP1 and AMPK (Fig. 3f). Given the downregulation of AMPK-α1, we hypothesized that mitophagy induced by CRL4 knockdown caused AMPK-α1 protein degradation. Therefore, we depleted CRL4 in OCCs following treatment with the indicated concentration of bafilomycin A1 (BafA1, autophagy inhibitor) and found that BafA1 significantly abrogated the decrease in AMPK-α1 protein levels (Supplementary Fig. 4g, h). To test whether CRL4 E3 ligase affects the cellular level of AMPK-α1, we overexpressed CRL4 in A2780CP cells and found that the endogenous protein level of AMPK-α1 barely changed (Supplementary Fig. 4i). These data suggest that CRL4^CUL4A/DDB1^ knockdown induced mitochondrial fission or even fragmentation, as well as mitochondrial translocation of DRP1, accompanied by Ser637 dephosphorylation of DRP1. Taken together, these results suggest that endogenous DRP1 plays an essential role in mediating mitochondrial fission in response to CRL4^CUL4A/DDB1^ depletion in OCCs.

### Knockdown of CRL4^CUL4A/DDB1^ induced autophagy and impaired lysosome degradation in cisplatin-resistant OCCs

**Fig. 4:**
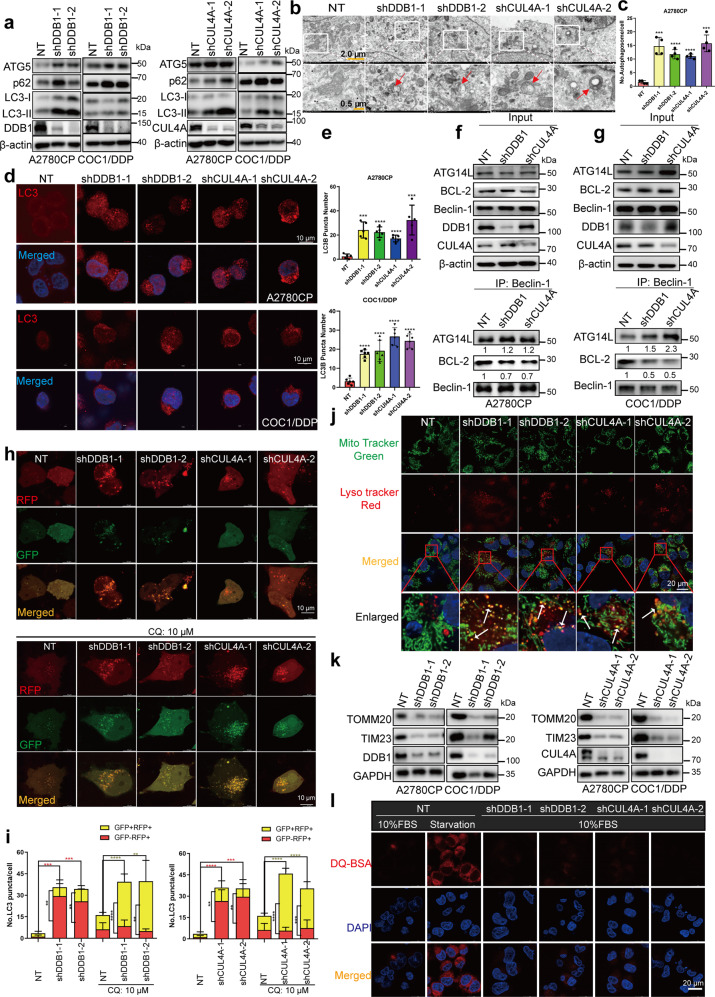
CRL4 knockdown induces autophagy in cisplatin resistance OCCs. **a** Immunoblotting analysis of LC3B, ATG5, and p62 expression in (left panel) A2780CP or (right panel) COC1/DDP cells with DDB1 or CUL4A knockdown. **b** Representative images of the transmission electron microscope in A2780CP cells treated as in (**a**). **c** Quantification of the LC3 puncta number in (**b**) (***p < 0.001, ****p < 0.0001, *t*-test). **d** Immunofluorescence analysis of LC3 in (upper panel) A2780CP and (lower panel) COC1/DDP cells with or without CRL4 knockdown. Scale Bar: 10 μm. **e** Quantification of autophagic vesicles in (**d**). Data represent mean ± SEM with three replicates, **p < 0.01, ***p < 0.001, ****p < 0.0001. **f**, **g** The interaction between BECN1 and BCL2, ATG14L was detected by Co-IP assay in **f** A2780CP and **g** COC1/DDP cells with CRL4 knockdown. **h** Immunofluorescence analysis of CRL4-knockdown A2780CP cells transiently transfected with tandem mRFP-GFP-tagged LC3B, followed by treatment with 10 μM chloroquine for another 24 h. Scale Bar: 10 μm. **i** Quantification of the ratio of autolysosome (AL, red puncta) versus autophagosome (AP, yellow puncta). *p < 0.05, **p < 0.01; ***p < 0.001, *t*-test. **j** Colocalization of mitochondria and autolysosomes analyzed by staining A2780CP cells with Lyso-Tracker Red and MitoTracker Green. Red indicates staining with Lyso-Tracker Red, green represents staining with MitoTracker Green, and yellow stands for the two colors merged together. Yellow puncta were counted as mitochondria having autolysosomes. White arrows indicate colocalization points. Scale bar: 10 μm. **k** Immunoblotting of mitochondrial outer membrane protein Tomm20 and inner membrane protein Tim23 in A2780CP or COC1/DDP cells transfected with lentivirus carrying shRNA targeting CUL4A (right panel) or DDB1 (left panel). GAPDH was used as a loading control for Tomm20, Tim23, DDB1, and CUL4A. Representative images were selected from three independent experiments. *p < 0.05, **p < 0.01; ***p < 0.001, *t*-test. **l** Representative images of CRL4 knockdown OCCs incubated with BODIPY-conjugated bovine serum (DQ-BSA, red) for 1 h, or with serum- and glucose-free medium (starvation). Scale bar: 20 μm

The elimination of whole mitochondria is accomplished by a selective form of autophagy, named mitophagy.^50^ Mitophagy is a form of mitochondrial quality control that removes poorly functioning mitochondria.^51^ As the results above confirmed mitochondrial dysfunction in response to CRL4^CUL4A/DDB1^ knockdown, we investigated whether CRL4^CUL4A/DDB1^ depletion induced mitophagy in cisplatin-resistant OCCs. Our results demonstrated that CRL4^CUL4A/DDB1^ downregulation promoted the conversion of LC3-I to lipidated LC3-II along with an increase in endogenous LC3 puncta (Fig. 4a–e). This suggested that autophagy was involved in mediating cisplatin-resistant OCCs. Of interest, we observed accumulation of the autophagy-specific substrate p62 (SQSTM1) in cisplatin-resistant CRL4^CUL4A/DDB1^ knockdown OCCs along with increased LC3-II levels (Fig. 4a). Additionally, depletion of CRL4^CUL4A/DDB1^ considerably increased p62 transcription, as shown in Supplementary Fig. 5a, b. Consistently, significant accumulation of autophagic vesicles and LC3-II puncta was also observed in CRL4-depleted cisplatin-resistant OCCs by transmission electron microscopy imaging, confirming that CRL4^CUL4A/DDB1^ knockdown increased the level of endogenous LC3 puncta (Fig. 4b, c). Depletion of CRL4^CUL4A/DDB1^ obviously increased the endogenous LC3 puncta, detected by immunofluorescence assay (Fig. 4d, e). Moreover, we observed that MLN4924 treatment also enhanced the accumulation of LC3-II in a dose- and time-dependent manner in OCCs (Supplementary Fig. 5c, d), and overexpressing CRL4^CUL4A/DDB1^ decreased the expression level of LC3-II (Supplementary Fig. 5e, f). These results suggest that CRL4^CUL4A/DDB1^ inhibits autophagy in cisplatin-resistant OCCs.

The initiation of autophagy requires the dissociation of Beclin-1 from BCL2 and subsequent binding with PIK3C3/VPS34 (PtdIns3K).^12,52^ We found that CRL4^CUL4A/DDB1^ depletion led to the disruption of the Beclin-1-BCL2 interaction and enhanced binding between Beclin-1 and ATG14L of the PIK3C3/VPS34 complex (Fig. 4f, g). An inhibitor of phosphatidylinositol-3-kinase,^53^ wortmannin, prominently counteracted the elevation of LC3B-II levels and the accumulation of endogenous LC3B puncta in CRL4^CUL4A/DDB1^ knockdown cisplatin-resistant OCCs (Supplementary Fig. 5g). These results suggest that knockdown of CRL4^CUL4A/DDB1^ initiates autophagy in OCCs. To determine whether the impaired autophagic flux is a result of failure in autophagosome-lysosome fusion or decreased capacity for autolysosomal degradation, a tandem mRFP-GFP-tagged LC3B construct was used. As shown in Fig. 4h, i, the number of LC3B puncta was markedly increased in CRL4^CUL4A/DDB1^ knockdown OCCs, with a large proportion of puncta exhibiting RFP+/GFP− signaling (autolysosomes) rather than RFP+/GFP+ signaling (autophagosomes). Conversely, chloroquine (CQ)-treated CRL4^CUL4A/DDB1^ knockdown OCCs showed a large proportion of puncta exhibiting RFP+/GFP+ signaling (autophagosomes) rather than RFP+/GFP− signaling (autolysosomes, Fig. 4i). To determine whether increased degradation of mitochondria occurred in CRL4^CUL4A/DDB1^ knockdown cisplatin-resistant OCCs, A2780CP, and COC1/DDP cells were double stained with Lyso-Tracker Red and MitoTracker Green fluorescent dyes to concurrently label lysosomes and mitochondria, respectively. The observation of small fragmented mitochondria was more evident in CRL4^CUL4A/DDB1^ knockdown cisplatin-resistant OCCs, whereas a tubular mitochondrial network was clearly distinct in NT control cells (Fig. 4j and Supplementary Fig. 5h). Compared with the NT control cells, the small fragmented mitochondria engulfed within the enlarged puncta (autolysosome) were broadly visualized in A2780CP and COC1/DDP cells depleted with CRL4^CUL4A/DDB1^, suggesting an enhanced mitophagy process, degrading damaged mitochondria within autolysosomes. Moreover, as shown in Fig. 4k, the mitochondrial outer membrane protein and inner membrane protein degradation induced by CRL4^CUL4A/DDB1^ knockdown was significantly increased in OCCs.

Additionally, the elevated LC3B-II levels may also be a consequence of impaired autophagic flux with defective degradation capacity.^54,55^ To verify this hypothesis, we introduced a highly self-quenched BODIPY-conjugated bovine serum albumin (DQ-BSA). Using starvation-treated A2780CP cells as a positive control,^54^ we observed decreased DQ-BSA fluorescence due to impaired capacity for proteolytic degradation in CRL4^CUL4A/DDB1^-knockdown cells, as shown in Fig. 4l. In agreement with these observations, GSK-621 or CCCP treatment for 24 h significantly promoted OCC autophagy as evidenced by western blotting, whereas immunofluorescence assay revealed decreased DQ-BSA fluorescence due to impaired capacity for proteolytic degradation in these treated cells (Supplementary Fig. 5i, j). These results suggest that CRL4^CUL4A/DDB1^ knockdown induced autophagy initiation and impaired the capacity for autolysosomal degradation in OCCs.

### Knockdown of CRL4^CUL4A/DDB1^ stimulates mitophagy via the Parkin-PINK1 pathway in cisplatin-resistant OCCs

**Fig. 5:**
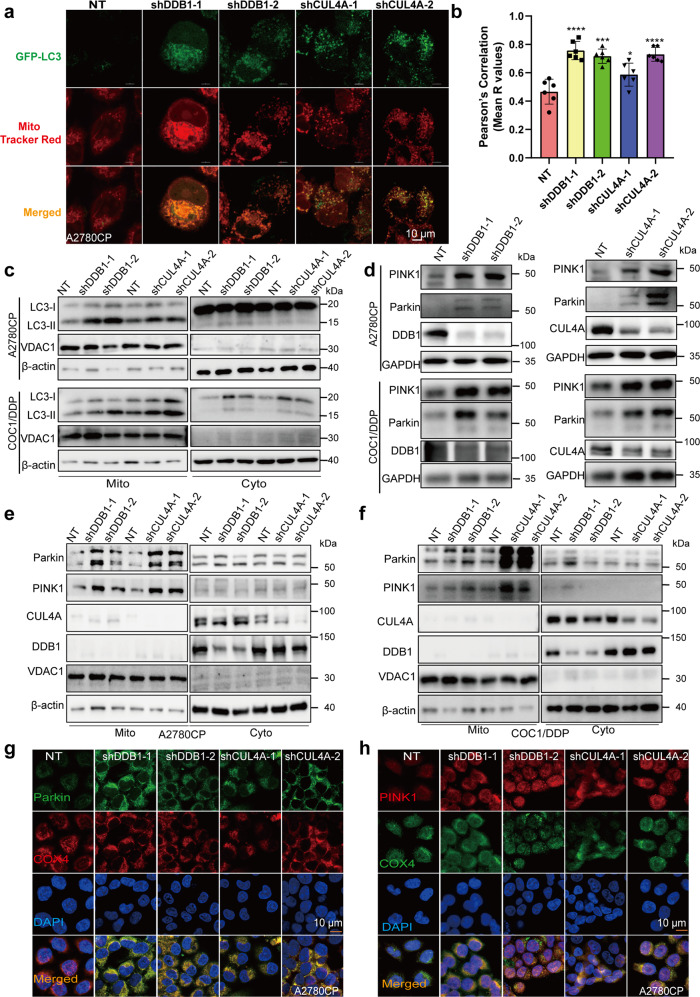
Knocking down CRL4^CUL4A/DDB1^ stimulates mitophagy by inducing mitochondrial Parkin translocation in cisplatin-resistant OCCs. **a** The colocalization of mitochondria and autophagosome was determined by immunofluorescence staining. Briefly, A2780CP cells were observed 24 h after CRL4^CUL4A/DDB1^ was knocked down, followed by staining with GFP-LC3 plasmids for 24 h and staining with MitoTracker Red for 30 min. Scale Bar:10 μm. **b** Graphs show the Pearson’s R values using whole cells by Image J. (*n* = 4–6 fields). **c** Immunoblotting analysis of LC3 in the cytosolic (Cyto) and mitochondrial (Mito) fractions of A2780CP (upper panel) and COC1/DDP (lower panel) cells with or without CRL4^CUL4A/DDB1^ knockdown. **d** Western blot analysis of Parkin, PINK1, DDB1, and CUL4A protein levels in A2780CP and COC1/DDP cells with or without CRL4^CUL4A/DDB1^ knockdown. **e**, **f** Immunoblotting analysis of Parkin and PINK1 in the cytosolic (Cyto) and mitochondrial (Mito) fractions of (left panel) A2780CP and (right panel) COC1/DDP cells with or without CRL4^CUL4A/DDB1^ knockdown. **g** Immunofluorescence analysis of the colocalization of endogenous Parkin and COX4 in A2780CP cells with or without CRL4^CUL4A/DDB1^ knockdown. Scale Bar:10 μm. **h** Immunofluorescence analysis of the colocalization of endogenous COX4 and PINK1 in A2780CP cells with or without CRL4^CUL4A/DDB1^ knockdown. Scale Bar:10 μm

Mitophagy is a form of mitochondrial quality control that removes poorly functioning mitochondria.^51^ As the results above confirmed mitochondrial dysfunction in response to CRL4^CUL4A/DDB1^ knockdown, we investigated whether CRL4^CUL4A/DDB1^ depletion induced mitophagy in cisplatin-resistant OCCs. We found that CRL4^CUL4A/DDB1^ knockdown or MLN4924 treatment promoted the conversion of LC3-I to lipidated LC3-II. To determine whether mitochondria were engulfed by autophagosomes, we first analyzed the colocalization of autophagosomes and mitochondria. The results showed that the knockdown of CRL4^CUL4A/DDB1^ significantly increased the colocalization of autophagosomes with mitochondria, as evidenced by the merged fluorescent signaling of GFP-LC3 and MitoTracker (Fig. 5a, b). In addition, western blot also showed that the knockdown of CRL4^CUL4A/DDB1^ considerably promoted the conversion of LC3-I to lipidated LC3-II in the mitochondrial distribution in OCCs (Fig. 5c). Recent studies have identified the PINK1/Parkin pathway as a key signaling pathway mediating mitophagy in mammalian cells.^56^ Thus, we detected the expression of Parkin and PINK1 in CRL4-depleted OCCs. As shown in Fig. 5d, the knockdown of CRL4^CUL4A/DDB1^ enhanced the expression of PINK1 and Parkin in OCCs, whereas overexpression of CRL4^CUL4A/DDB1^ barely changed these effects (Supplementary Fig. 6a, b). Translocation of Parkin to mitochondria is a hallmark of mitophagy.^57^ We then examined the mitochondrial translocation of Parkin in OCCs by analyzing cellular fractions. As expected, we observed enriched Parkin in the mitochondrial fraction in OCCs with CRL4^CUL4A/DDB1^ knockdown (Fig. 5e, f). These results were further supported by the increased colocalization between the Cytochrome C oxidase subunit 4 isoform 1 (COX4) and Parkin/PINK1 proteins in mitochondria in CRL4^CUL4A/DDB1^ knockdown OCCs (Fig. 5g, h). Additionally, growing evidence indicates that FUN14 domain-containing protein 1 (FUNDC1) could be a mitophagy receptor controlled by phosphorylation by ULK1 on S17,^58^ and NIX serves as a selective autophagy receptor that facilitates the recruitment of LC3/GABARAP for mitochondrial clearance.^59^ Our results indicated that CRL4^CUL4A/DDB1^ depletion had no obvious effect on BNIP3/NIX and FUNDC1 in OCCs (Supplementary Fig. 6c, d). Collectively, these data suggest that CRL4^CUL4A/DDB1^ depletion stimulates mitophagy by inducing mitochondrial Parkin/PINK1 translocation in OCCs.

### Knockdown of CRL4^CUL4A/DDB1^ inhibited OCC growth by inducing mitophagy

**Fig. 6:**
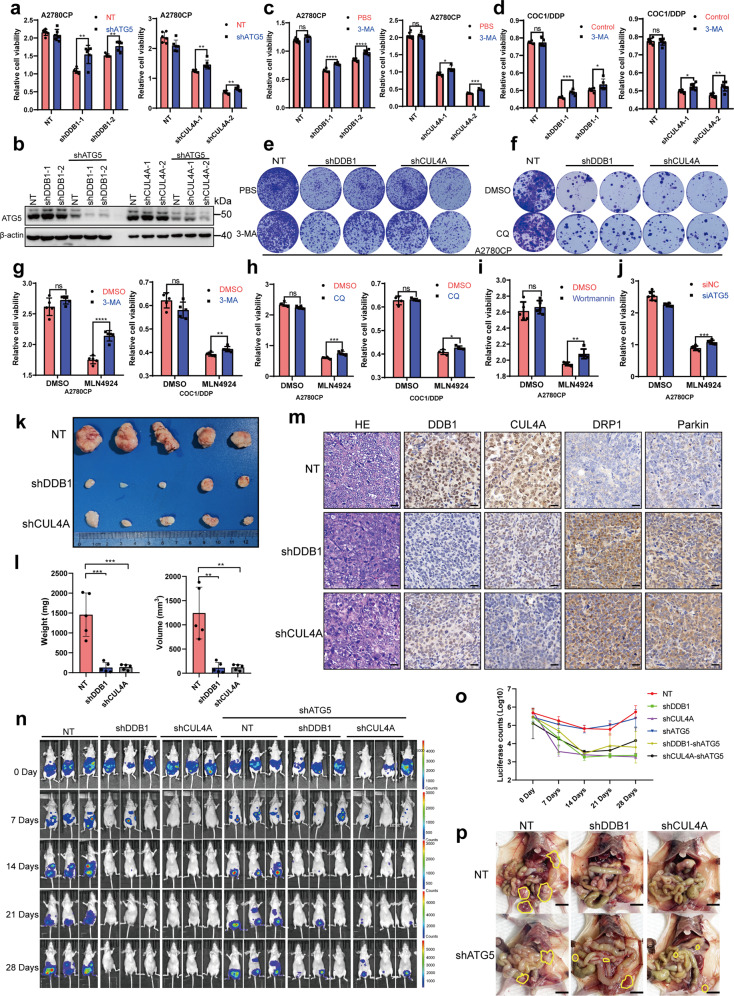
Knocking down CRL4^CUL4A/DDB1^ inhibits OCC growth by inducing mitophagy. **a** Cell proliferation determined by CCK8 assay in A2780CP cells after CRL4^CUL4A/DDB1^ knockdown with or without shATG5. **b** Verification of ATG5 knockdown in A2780CP cells using shRNA. **c**, **d** Cell proliferation determined by CCK8 assay in A2780CP (**c**) and COC1/DDP (**d**) cells after CRL4^CUL4A/DDB1^ knockdown with or without 1 mM of 3-MA treatment for 36 h. **e** Cell proliferation determined by colony formation assay in A2780CP cells (10,000 cells) after CRL4^CUL4A/DDB1^ knockdown with or without 1 mM of 3-MA treatment for 36 h. **f** Cell proliferation determined by colony formation assay in A2780CP cells (3000 cells) after CRL4^CUL4A/DDB1^ knockdown with or without 4 µM of CQ treatment for 24 h. **g** Cell proliferation determined by CCK8 assay in (left panel) A2780CP and (right panel) COC1/DDP cells after treatment with 0.5 µM MLN4924 for 24 h with or without 1 mM of 3-MA treatment for 36 h. **h** Cell proliferation determined by CCK8 assay in (left panel) A2780CP and (right panel) COC1/DDP cells after treatment with 0.5 µM MLN4924 for 24 h with or without 4 µM of CQ treatment for 24 h. **i** Cell proliferation determined by CCK8 assay in A2780CP cells after treatment with 0.5 µM MLN4924 for 24 h with or without 10 µM of Wortmannin treatment for 24 h. **j** Cell proliferation determined by CCK8 assay in A2780CP cells after ATG5 knockdown with or without 0.5 µM of MLN4924 treatment for 24 h. **k** Representative image of isolated A2780CP tumor xenografts from mice in cohorts. Female immunodeficient nude mice received a subcutaneous injection of 5 × 10^6^ A2780CP cells containing either shDDB1/shCUL4A or negative control constructs (NT). End-point tumors isolated after four weeks of growth are shown. **l** Quantification of (left panel) total tumor weight and (right panel) tumor volume for A2780CP xenografts (*n* = 5 per group). Statistical significance was determined using a *t*-test (*p < 0.05). **m** Immunohistochemical staining analysis of hematoxylin-eosin stain, DDB1, CUL4A, DRP1, and Parkin staining in NT or shCUL4A/DDB1 tumors. **n** Representative ventral bioluminescent images of nude mice injected with A2780CP-luc-puro cells transduced with indicated shRNA constructs on days 0, 7, 14, 21, and 28. **o** Longitudinal ventral bioluminescence measurement for all mice injected with A2780CP-luc-puro transduced with indicated shRNA. Data represent mean total counts (photons/sec) for each group ± SEM. (*n* = 6 mice per group). **p** Representative end point necropsy showing differential tumor size and distribution in mice injected with A2780CP cells. Scale bar, 1 cm. Visible tumors are circled in yellow. (For all panels in this figure, *p < 0.05, **p < 0.01, ***p < 0.001, ****p < 0.0001, *t*-test)

As reviewed by Youle and Narendra, mitophagy regulates mitochondrial number and maintains quality control by selectively removing damaged mitochondria.^51^ Aberrant mitophagy is presumed to promote cell death by disturbing mitochondrial homeostasis.^60^ Our previous study also illustrated that CRL4^CUL4A/DDB1^ E3 ubiquitin ligase regulated OC drug resistance by targeting the antiapoptotic protein BIRC3.^35^ Indeed, the suppression of CRL4 knockdown-induced mitophagy by ATG5 knockdown resulted in alleviation of CRL4 knockdown-induced cytotoxicity in OCCs (Fig. 6a, b). A similar increase in both cell viability and clonogenic survival was also observed in CRL4 knockdown OCCs in combination with 3-MA treatment (Fig. 6c–e), suggesting that mitophagy induction, rather than other potential effects of CRL4^CUL4A/DDB1^ knockdown, contributed to the dysregulation in OCCs. Consistently, a significant increase in clonogenic survival was also observed in CRL4 knockdown OCCs in combination with CQ treatment (Fig. 6f). Notably, both ATG5 knockdown and autolysosome inhibition (3-MA, wortmannin or CQ treatment) partially reversed MLN4924-induced cell proliferation inhibition in OCCs, as shown in Fig. 6g–j. Thus, these data suggest that CRL4^CUL4A/DDB1^ downregulation induces cytotoxic mitophagy in OCCs.

### CRL4^CUL4A/DDB1^ regulates the seeding and growth of peritoneal tumors

To evaluate the anticancer effect of CRL4^CUL4A/DDB1^ knockdown in vivo against OC, a tumor xenograft model was performed by subcutaneously inoculating OC cells into nude mice. The results revealed that the knockdown of CRL4^CUL4A/DDB1^ markedly decreased the size and weight of OC xenografts (Fig. 6k, l). As expected, CRL4^CUL4A/DDB1^ knockdown resulted in stronger DRP1 and Parkin staining compared with the NT control group (Fig. 6m). We also used an intraperitoneal tumor xenograft model to further validate the important roles of CRL4^CUL4A/DDB1^ and mitophagy in ovarian tumor progression. Loss of CRL4^CUL4A/DDB1^ significantly inhibited peritoneal seeding and tumor growth of A2780CP cells, whereas ATG5 depletion partially protected A2780CP cells from cell death, as measured by bioluminescence (Fig. 6n, o). Consistently, terminal necropsy verified a general disappearance in the size of metastatic nodules in the viscera, peritoneal wall, and omentum, but tumor growth obviously recovered with ATG5 depletion (Fig. 6p). Thus, these results demonstrate a role for mitophagy of CRL4 downregulation in the establishment and growth of peritoneal tumors.

**Fig. 7:**
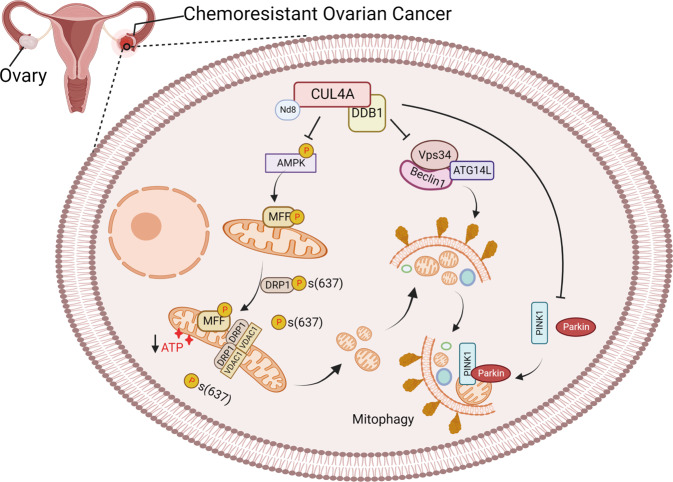
The schematic diagram showing the underlying mechanisms involved in mitophagy repression by CRL4^CUL4A/DDB1^ in chemoresistant ovarian cancer. Downregulation of CRL4^CUL4A/DDB1^ promotes mitophagy by modulating the Parkin-PINK1 axis, MFF phosphorylation, DRP1^Ser637^ dephosphorylation, as well as the interaction between DRP1 and VDAC1, eventually inducing mitochondrial fission and selectively removing fragmented mitochondrial in chemoresistant ovarian cancer cells. [created with BioRender.com (https://biorender.com/)]

In summary, our data suggest that CRL4^CUL4A/DDB1^ is crucial for mitochondrial function in cisplatin-resistant OCCs. Depletion of CRL4^CUL4A/DDB1^ enhanced mitochondrial fission by mediating DRP1 expression and its phosphorylation at Ser637 (Fig. 7). The dysfunctional and fragmented mitochondria were degraded by mitophagy via the Parkin/PINK1 pathway (Fig. 7). Notably, inhibition of autophagy through shATG5 and autolysosome inhibition (3-MA, wortmannin or CQ treatment) partially reversed CRL4^CUL4A/DDB1^ knockdown- or MLN4924-induced cell proliferation inhibition in OCCs. Thus, these results suggest that targeting CRL4^CUL4A/DDB1^ and mitophagy may be a novel strategy to overcome drug resistance in OC.

## Discussion

Primary systemic platinum-based chemotherapy acts as the first-line treatment for a variety of malignancies, including ovarian cancer (OC). Although cisplatin-based treatment is still effective for most OC patients, almost all patients eventually develop recurrence and acquire progressive resistance over time.^61,62^ Growing evidence suggests that dysregulation of mitochondrial dynamics plays important role in human carcinogenesis, including in OC.^63^ Likewise, Kong et al. have shown that mitochondrial dysregulation is involved in the increasing degree of chemoresistance in gynecologic cancers, including OC.^64,65^ Therefore, a better understanding of the molecular mechanisms underlying cisplatin resistance and mitochondrial dynamics is essential for clinical therapy development in OC.

In this study, we discovered a novel regulatory mechanism of CRL4^CUL4A/DDB1^ E3 ligase in OC chemotherapy resistance. We found that CRL4^CUL4A/DDB1^ was significantly upregulated in cisplatin-resistant OCCs and that knockdown of CRL4^CUL4A/DDB1^ inhibited cell proliferation, dysregulated mitochondrial functions, and induced mitophagy in cisplatin-resistant OCCs. Importantly, we identified a CRL4^CUL4A/DDB1^ target, DRP1, which is a mitochondrial fission protein. DRP1 upregulation in CRL4^CUL4A/DDB1^ knockdown cells correlated with enhanced mitochondrial fission and decreased p-DRP1^Ser637^ (Fig. 3). We also revealed a mechanistic link between CRL4^CUL4A/DDB1^ and the Parkin/PINK1 signaling pathway in mediating mitophagy in OCCs (Fig. 5).

Historically, the importance of mitochondria in tumor progression has been neglected for a long time. Mitochondria are often regarded as cellular powerhouses due to their ability to efficiently produce adenosine triphosphate (ATP) to ensure normal cellular physiological functions. Recently, the role of mitochondria in cancer has been proven experimentally in both human and mouse studies.^66–68^ Increasing evidence suggests that cancer progression and chemoresistance are coupled with mitochondrial dysfunction, thus, interfering mitochondrial functions may be utilized in anticancer therapies via drugs and genetic approaches.^9^ Research by Wang et al. showed that mitochondrial dysfunction regulates the ROS-activated GCN2-eIF2α-ATF4-xCT pathway, enhancing cisplatin resistance in human gastric cancer cells.^69^ In this study, we showed that irregular expression of CRL4^CUL4A/DDB1^ facilitated mitochondrial dysfunction, including decreased mitochondrial membrane potential, decreased ATP synthesis, and diminished oxygen consumption rate of mitochondria in cisplatin-resistant OCCs. This downregulation was strongly consistent with the inhibition of cell proliferation.

The multifaceted involvement of mitochondria is coupled with these dynamic changes in mitochondrial morphology in cell biology. Accordingly, mitochondrial dynamics have been implicated not only in metabolism but also in several complex cellular processes, such as the regulation of cell pluripotency, differentiation, senescence, division, and cell death.^70^ Thus, we examined key posttranslational modifications of proteins involved in mitochondrial dynamics, such as proteins required for mitochondrial fusion, OPA1, MFN1, and MFN2, as well as proteins required for mitochondrial fission, DRP1 and Fis1. Our results revealed that DRP1 upregulation in CRL4^CUL4A/DDB1^ knockdown cells correlated with the boosted ring-shaped mitochondrial structure. However, no significant changes were found in other proteins regulating mitochondrial morphology. We have gone one step further than only looking at the expression of DRP1 and found some surprisingly large effects. Studies have revealed that dephosphorylation of DRP1 at Ser637 by calcineurin appears to increase the mitochondrial recruitment of DRP1, in turn promoting mitochondrial fission.^71,72^ Specifically, DRP1^Ser637^ dephosphorylation significantly increased in CRL4^CUL4A/DDB1^ knockdown OCCs, which attenuated the inhibition of DRP1 activation and resulted in mitochondrial fission. Likewise, CRL4 knockdown enhanced the interaction between DRP1 and VDAC1. This was consistent with the diminished level of AMPK phosphorylated DRP1^Ser637^ in CRL4^CUL4A/DDB1^ knockdown cells (Fig. 3). It has been reported that AMPK promotes the clearance of dysfunctional mitochondria by phosphorylating mitochondrial fission factor (MFF), regulating DRP1 and activating ULK1, which acts as an upstream kinase in autophagy and promotes mitophagy.^73^ As expected, CRL4 knockdown enhanced the level of AMPK-dependent MFF phosphorylation and mitochondrial recruitment of DRP1 by phosphorylated MFF, translocating DRP1 to mitochondrial and initiating mitochondrial fission (Fig. 3). Whether ULK1 may be involved in the mitochondrial division process of cisplatin-resistant OCCs still needs further confirmation in future studies.

Mitophagy, defined as the selective engulfment and clearance of damaged mitochondria, is crucial for cellular homeostasis that precludes superfluous production of cytotoxic reactive oxygen species from dysfunctional mitochondria.^74^ Growing evidence shows that defective and excessive mitophagy accelerates a number of pathologies, including metabolic diseases, liver injury, diabetes and Parkinson’s disease.^75^ The mitochondria-targeted Parkin and PINK1 are well-recognized and synergistically committed to mitophagy of defective mitochondria in a cell.^74^ In this manuscript, we demonstrated that CRL4^CUL4A/DDB1^ knockdown significantly boosted the expression of Parkin and PINK1 and enhanced the mitochondrial translation of Parkin and PINK1 in cisplatin-resistant OCCs (Fig. 5). Research by Sven Geisler and colleagues showed that PINK1/Parkin-mediated mitophagy was dependent on VDAC1 and p62/SQSTM1.^76^ As expected, CRL4 downregulation in OCCs promoted p62 expression (Fig. 4a and Supplementary Fig. 5a, b). The accumulation of misfolded proteins induced by CRL4 knockdown led to aberrant p62 expression, which may disrupt the balance of mitophagy, in turn forming a vicious cycle. Teranishi et al. recently showed the mechanism where the CUL4A-DDB1-WDFY1 E3 ubiquitin ligase complex mediated K48-linked polyubiquitination of LAMP2 (lysosomal associated membrane protein 2) upon lysosomal damage, which recruited the autophagic machinery.^77^ LAMP1/2 are distributed among autophagic and endolysosomal organelles, where LAMP1 is often used as a lysosome marker and LAMP1-positive organelles are often referred to as lysosomal compartments.^78^ *CUL4A* is located closely with *LAMP1* on the chromosome, their expression levels are also positively correlated in ovarian cancer tumor tissues, suggesting they may have cooperative functions. Therefore, it is possible that CRL4^CUL4A/DDB1^ depletion impairs lysosomes by interrupting LAMP1/2 functions. Further research is needed to provide a clearer and more objective conclusion.

In the present study, our findings unveiled the mechanism whereby CRL4^CUL4A/DDB1^ E3 ligase regulated mitophagy biogenesis in cisplatin-resistant OCCs, suggesting that CRL4^CUL4A/DDB1^ may serve as a novel anticancer target in cisplatin-resistant OC. MLN4924, an inhibitor of CRL4^CUL4A/DDB1^ activity, may also be an effective drug to treat OC in combination with cisplatin or its derivatives. Targeting E3 ubiquitin ligases for anticancer therapies is one of the hot topics in current cancer research. Therefore, our discovery will shed light on drug development based on mitophagy regulation and provide a clue for developing promising drugs to overcome chemoresistance in OC therapy.

## Materials and methods

### Ethics statements

Tissue microarray chips consisting of ovarian tumor tissue specimens were purchased from Outdo Biotech, Ltd. (Shanghai, China). The specimens were collected from the National Human Genetic Resources Sharing Service Platform (Platform No. 2005DKA21300), with approval from the Institutional Ethics Committee of Outdo Biotech, Ltd. (Approval No. SHYJS-CP-1804010).

The animal experiments were performed in strict accordance with the People’s Republic of China Legislation Regarding the Use and Care of Laboratory Animals. All protocols used in this study were approved by the Institutional Animal Care and Treatment Committee of Sichuan University in China. (Approval No. 20220218005).

### Cell culture and reagents

A2780 and A2780CP OC cell lines were cultured in DMEM (Cytiva, SH30243.01, USA) supplemented with 10% fetal bovine serum (Excell Bio, FSP500, HK), 100 U/mL penicillin, and 100 μg/mL streptomycin (Beyotime, C0222, China). The cisplatin-sensitive human ovarian adenocarcinoma cell line COC1 and the cisplatin-resistant cell line COC1/DDP were obtained from Shengtao Zhou’s Lab of West China Second Hospital. Both cell lines were cultured in RPMI (Cytiva, SH30809.01, USA) supplemented with 10% fetal bovine serum (Excell Bio, FSP500, HK), 100 U/mL penicillin, and 100 μg/mL streptomycin (Beyotime, C0222, China). The cell culture was maintained in a humidified incubator at 37 °C under 5% CO_2_. Cisplatin was purchased from J&K Scientific Ltd. (15663-27-1, China). Wortmannin (MCE, HY-10197, USA) and 3-methyladenine (3-MA) (MCE, HY-19312, USA) were purchased from MedChemExpress.

### Antibodies

The following antibodies were used in this study: anti-CUL4A (Proteintech, 14851-1-AP, 1:2000), anti-DDB1 (HuaBio, ET1706-22, 1:2000), anti-DRP1 (C-Terminal, Proteintech, 12957-1-AP, 1:2000), anti-p62 (Proteintech, 18420-1-AP, 1:2000), anti-LC3 (Proteintech, 14600-1-AP, 1:3000), anti-ATG5 (Proteintech, 10181-2-AP, 1:2000), anti-ATG14L (Proteintech, 19491-1-AP, 1:2000), anti-BCL-2 (Proteintech, 12789-1-AP, 1:1000), anti-MFF (Proteintech, 17090-1-AP, 1:1000), anti-phospho-MFF^Ser172/Ser146^ (Affinity, AF2365, 1:1000) anti-Beclin-1 (Proteintech, 11306-1-AP, 1:1000), anti-phospho-DRP1-Ser616 (CST, #3455, 1:1000), anti-phospho-DRP1-Ser637 (ABclonal, AP0812, 1:1000), anti-phospho-AMPKα-Thr172 (CST, #2535 1:1000), anti-VDAC1 (Proteintech, 10866-1-AP, 1:2000), anti-AMPK alpha 1 (HuaBio, ET1608-40, 1:1000), anti-Parkin (HuaBio, ET1702-60, 1:1000), anti-PINK1 (HuaBio, ER1706-27, 1:1000), anti-BNIP3 (Abclonal, A5683, 1:1000), anti-BNIP3L (Abclonal, A6283, 1:1000), anti-FUNDC1 (Abclonal, A16318, 1:1000), anti-PGC-1α (Abclonal, A12348, 1:1000), anti-Tomm20 (Proteintech, 11802-1-AP, 1:2000), anti-Tim23 (Proteintech, 11123-1-AP, 1:2000). GAPDH (ZSGB-Bio, TA-08, 1:5000) and β-actin (ZSGB-Bio, TA-09, 1:1000) were used as sample loading controls. Relative quantification of protein expression was conducted using Image J software (NIH, USA).

### Mitochondrial cell stress assay

Oxygen consumption rate (OCR) measurements were performed by using a Seahorse XFp Analyser (Agilent Technologies, USA) in OCCs. Briefly, cells were seeded in an XF24 cell culture plate (40,000 cells/well). Cell culture medium was replaced with serum-free XF medium supplemented with 25 mmol/L glucose and 1 mmol/L sodium pyruvate. Subsequently, the cells were incubated for 1 h in a non-CO_2_ 37 °C incubator prior to the start of the assay. The calibration solution was preincubated with cartridges equipped with oxygen- and pH-sensitive probes overnight in a non-CO_2_ 37 °C incubator. Then, the cells were placed in an XF24 Extracellular Flux Analyser with cartridges equipped with probes at the time of measurement. The oxygen consumption rate (OCR) was examined in a time course before and after injection of the compounds, including oligomycin (1 μM final concentration), FCCP [carbonyl cyanide-4-(trifluoromethoxy) phenylhydrazone; 1 μM final concentration], and antimycin A + rotenone (1.5 μM each final concentration).

### Adenosine triphosphate (ATP) content assay

The intracellular ATP concentration was measured using an ATP assay kit (Beyotime, S0026, China). Cells (1 × 10^6^) were infected with shCRL4-containing lentivirus for 24 h and continued to culture for another 24 h at 37 °C. Cells were then lysed and centrifuged at 12,000 × *g* for 5 min at 4 °C. Luciferase intensity was measured on a multimode detection platform in luminance mode. The content of ATP was calculated according to the following formula: ATP content = ATP concentration/protein concentration.

### Immunohistochemical (IHC) staining assay and tissue microarray analysis

Tissue microarray chips consisting of the ovarian tumor tissue specimens (Supplementary Tables S1, S2) were purchased from Outdo Biotech, Ltd. (Shanghai, China). Hematoxylin-eosin (H&E) staining was performed by following the routine method and IHC of TMA chips was performed following standard IHC staining protocol with primary antibodies against DDB1 (1:30,000) and CUL4A (1:5000). The assignment of the positive-staining score was based on the following standard: the percentage of staining-positive cells (A) by the intensity (B: 0, negative; 1, weakly positive; 2, positive; 3, strongly positive). The final score for each case was calculated as A × B. Mann–Whitney *U*-method was used for statistical analysis.

Tumor xenografts were formalin-fixed, paraffin-embedded, and sectioned according to the standard protocol.^79^ Slides were incubated with primary antibodies (anti-CUL4A 1:200, anti-DDB1 1:200, anti-DRP1 1:400 and anti-Parkin 1:50) at 4 °C overnight and were subjected with secondary antibody (1:250) for 40 min at 25 °C . Then, the sections were performed with DAB chromogen and counterstained with hematoxylin. All image were visualized using a Leica DM 2000 microscope.

### Immunofluorescence

Cells were plated on glass coverslips in 24-well plates. After treatment, the cells were fixed with 4% paraformaldehyde in PBS for 30 min. After washing with PBS, the cells were permeabilized with 0.4% Triton X-100 and blocked with 5% goat serum for 30 min. The indicated primary antibodies were incubated with the cells overnight at 4 °C, followed by incubation with secondary antibodies (CoraLite 488-conjugated AffiniPure goat anti-mouse IgG (H + L) or CoraLite 594-conjugated goat anti-rabbit IgG (H + L)) at 37 °C for 1 h. After staining the nuclei with DAPI for 10 min, images were captured with a NiKon STORM Super-Resolution Microscope (Nikon A1 R+, Nikon, Japan). For the mitoTracker Red/Green (or Lyso-Trakcer Red) assay, cells were stained with MitoTracker Red (Beyotime, C1049B, China), MitoTracker Green (Beyotime, C1048, China) and Lyso-Trakcer Red (Beyotime, C1046, China). Cells were then counterstained with Hoechst 33342 (RiboBio, C10310-3, China) according to the manufacturer’s protocol. Images were taken using laser confocal microscopy as soon as possible.

For the DQ-BSA assay, cells were pre-probed with DQ-BSA Red (10 μg/mL; Thermo Fisher Scientific, D-12051) for 1 h. Then, cells were counterstained with Hoechst 33342 (RiboBio, C10310-3, China) according to the manufacturer’s protocol and were fixed with 4% paraformaldehyde in PBS for 30 min. Images were taken using laser confocal microscopy as soon as possible. For mitochondrial morphology assay, cells (1 × 10^5^) were plated into Confocal dishes (Bio SORFA, China) for 24 h and then were pre-probed with mitochondrial Tracker green (Beyotime, C1048, China) for 45 min at 37 °C. Then cells were washed twice with PBS. Images were taken using laser confocal microscopy as soon as possible. The mitochondrial footprint was analyzed by using the mitochondrial Network Analysis (MiNA) toolset. The mitochondrial footprint of each single cell was analyzed.^80^

### RNA extraction and quantitative real-time PCR

Total cellular RNA was isolated by using a Cell Total RNA Isolation Kit (Foregene, RE-03111, China). Reverse transcription was performed using the PrimeScript™ RT reagent Kit (Takara, RR036A, Japan) following the manufacturer’s protocol. Quantitative PCR using SYBR Green Supermix (Novoprotein, E096, China) was performed using a CFX96 Real-Time PCR System (Bio-Rad, USA). The relative expression levels of target genes were normalized to 18S ribosomal N5. The custom-made primers for genes tested in RT-PCR analysis are shown in Supplementary Table S3.

### Cell viability assay and colony formation assay

Cell viability was assessed by CCK8 (cell counting kit) assay. Briefly, cells were seeded in 96-well plates (3–4 × 10^3^ cells/well) with the indicated treatment. CCK8 reagent was then added to each well for 1–2 h at 37 °C. The OD value was measured at 450 nm in a microplate reader epoch2 (Bio-Tek, USA). For the colony formation assay, OCCs with shCUL4A/shDDB1 knockdown or scrambled shRNA (nontarget, NT) were seeded in a 12-well plate and treated with 3-MA or chloroquine (CQ) the next day. Twelve days later, the cells were fixed with 4% paraformaldehyde in PBS for 30 min and then stained with crystal violet for 30 min. The dishes were gently washed with ddH_2_O three times, and cell colonies were counted.

### Flow cytometric analysis

The mitochondrial membrane potential (MMP) was determined with a JC-1 assay kit (Beyotime, C2006, China). In addition, reactive oxygen species (ROS) were analysed using a ROS assay kit (Beyotime, S0033S, China). These experiments were performed based on the corresponding manufacturer’s instructions. Stained cells (>10,000) were examined by using a FACS flow cytometer (Beckman CytoFLEX, Beckman Coulter Life Sciences, USA). Flow Jo software was used to analyze the experimental data.

### RNA interference

siRNA targeting ATG5 and scrambled siRNA were synthesized by RiboBio (RiboBio, China). The siRNA sequences were as follows: Human ATG5 siRNA, 5′-GCAACUCUGGAUGGGAUUGTT-3′. The siRNA was transfected with Lipofectamine 3000 reagent (Invitrogen, #L300015, USA) for 48 h, according to the manufacturer’s protocol.

### Immunoblotting and immunoprecipitation

Whole-cell lysates were harvested and lysed in Radio-Immune Precipitation Assay (RIPA) lysis buffer and subjected to SDS-PAGE. Then, the proteins were transferred to PVDF membranes. After blocking with 5% nonfat milk blocking buffer for 1 h at room temperature, the target proteins were detected by specific antibodies, for 12 h at 4 °C. The PVDF membrane was then washed and probed with horseradish peroxidase-conjugated secondary antibodies for 2 h at room temperature. The PVDF membranes were washed again and then visualized by enhanced chemiluminescence (Millipore, USA).

### Transmission electron microscopy

A2780CP and COC1/DDP cells were fixed in 0.1% glutaraldehyde (Sigma, USA). Then, ultrathin sections were prepared using a Sorvall MT5000 microtome (DuPont Instruments, USA) after dehydration. And the samples were stained by lead citrate and/or uranyl acetate. Philips EM420 electron microscopy was used to analyze autophagic vesicles.

### Lentiviral transduction

CRL4^CUL4A/DDB1^-silenced OCCs were generated by lentiviral infection. HEK293T cells were co-transfected with lentiviral packaging plasmids, psPAX2 and pMD2.G, along with shCUL4A, shDDB1 (sequences shown in Supplementary Table S4) or the corresponding control shRNA pLKO.1 plasmid to produce lentivirus. Supernatant from each HEK293T culture was then collected at 24–72 h and filtered (0.45 μm filter). OCCs were subsequently transduced with lentiviral-containing supernatant and then selected with DMEM containing puromycin (2 μg/mL; Sigma-Aldrich, USA) for 48–72 h.

### Intraperitoneal tumor xenograft assay

The animal model was established as described previously.^81^ Briefly, cells (5 × 10^6^ A2780CP-luc-puro) were injected intraperitoneally into BALB/cA-nu mice (HFK Bioscience, China; 4–6-week-old females, six mice per group, randomly assigned for injection). Study numbers allow the detection of an effect size of 1.6 relative to the control with a power of 85% (*p* < 0.05 using Student’s *t*-test), assuming a tumor take rate of 80%. Tumor growth was monitored weekly (nonblinded) using the IVIS Spectrum in vivo imaging system (Perkin Elmer, USA) in the Frontiers Science Center for Disease-related Molecular Network for Molecular Imaging until shNT control mice achieved humane endpoints. Mice were injected with 200 μL d-Luciferin Potassium Salt (Biovision, 115144-35-9, USA) 15 min before imaging. Statistical significance was determined as described in the Statistical analysis section.

### Statistical analysis

All statistical analyses and graphics were performed using GraphPad 8 software. One-way ANOVA or Student’s *t*-test was used to analyze significant differences. All data were displayed as the mean ± SEM of at least three independent experiments. *p* < 0.05 was considered statistically significant.

## Supplementary information

Supplementary_Materials_Word

Supplementary Table S1

Supplementary Table S2

Supplementary Table S3

Supplementary Table S4

## Data Availability

All data were available within the manuscript, supplementary materials, or available from the corresponding author upon reasonable request.
