# Supplementary material for: Targeting CRL4 suppresses chemoresistant ovarian cancer growth by inducing mitophagy
Source: Signal Transduct Target Ther. 2022 Dec 9;7:388. doi: 10.1038/s41392-022-01253-y (PMC9731993; doi:10.1038/s41392-022-01253-y)
Supplement: Supplementary file 1 — Supplementary_Materials_Word [file 41392_2022_1253_MOESM1_ESM.docx]

Supplementary Materials for

**Targeting CRL4 suppresses chemoresistant ovarian cancer growth by inducing mitophagy**

Yang Meng^1^*, Lei Qiu^1^*, Xinyi Zeng^1,2^, Xiaoyan Hu^3^, Yaguang Zhang^1^, Xiaowen Wan^1^, Xiaobing Mao^1^, Jian Wu^1^, Yongfeng Xu^4^, Qunli Xiong^4^, Zhixin Chen^1^, Bo Zhang^1^, Junhong Han^1#^

Correspondence to: [hjunhong@scu.edu.cn](mailto:hjunhong@scu.edu.cn)

**This PDF file includes:**

Materials and Methods

Supplementary Figures. S1 to S6

**Other Supplementary Materials for this manuscript include the following:**

Supplementary Tables. S1 to S4

Supplementary Table S1. Patient information of tissue microarray for DDB1 staining.

Supplementary Table S2. Patient information of tissue microarray for CUL4A staining.

Supplementary Table S3. Primers used in the qPCR experiments.

Supplementary Table S4. Oligonucleotides sequences for shRNA used in this study.

**Materials and Methods**

**Immunofluorescence**

Cells were plated on glass coverslips in 24-well plates. After treatment, the cells were fixed with 4% paraformaldehyde in PBS for 30 min. After washing with PBS, the cells were permeabilized with 0.4% Triton X-100 and blocked with 5% goat serum for 30 min. The indicated primary antibodies were incubated with the cells overnight at 4 °C, followed by incubation with secondary antibodies (CoraLite 488-conjugated AffiniPure goat anti-mouse IgG (H+L) or CoraLite 594-conjugated goat anti-rabbit IgG (H+L)) at 37 °C for 1 h. After staining the nuclei with DAPI for 10 min, images were captured with a NiKon STORM Super-Resolution Microscope (Nikon A1 R+, Nikon, Japan). For the mito-Tracker Red/Green (or Lyso-Trakcer Red) assay, cells were stained with Mito-Tracker Green (Beyotime, C1048, China). Cells were then counterstained with Hoechst 33342 (RiboBio, C10310-3, China) according to the manufacturer’s protocol. Images were taken using laser confocal microscopy as soon as possible.

For DQ-BSA assay, cells were pre-probed with DQ-BSA Red (10 μg/mL; Thermo Fisher Scientific, D-12051) for 1 h. Then, cells were counterstained with Hoechst 33342 (RiboBio, C10310-3, China) according to the manufacturer’s protocol and were fixed with 4% paraformaldehyde in PBS for 30 min. Images were taken using laser confocal microscopy as soon as possible. For mitochondrial morphology assay, cells (1 × 10^5^) were plated into Confocal dishes (Bio SORFA, China) for 24 h and then were pre-probed with mitochondrial Tracker green (Beyotime, C1048, China) for 45 min at 37°C. Then cells were washed twice with PBS. Images were taken using laser confocal microscopy as soon as possible. The mitochondrial footprint was analyzed by using mitochondrial Network Analysis (MiNA) toolset. The mitochondrial footprint of each single cell was analyzed^1^.

**RNA extraction and quantitative real-time PCR**

Total cellular RNA was isolated by using a Cell Total RNA Isolation Kit (Foregene, RE-03111, China). Reverse transcription was performed using the PrimeScript™ RT reagent Kit (Takara, RR036A, Japan) following the manufacturer’s protocol. Quantitative PCR using SYBR Green Supermix (Novoprotein, E096, China) was performed using a CFX96 Real Time PCR System (Bio-Rad, USA). The relative expression levels of target genes were normalized to 18S ribosomal N5. The custom-made primers for genes tested in RT-PCR analysis shown in Supplementary Table 3.

**Cell viability assay**

Cell viability was assessed by CCK8 (cell counting kit) assay. Briefly, cells were seeded in 96-well plates (3-4×10^3^ cells/well) with the indicated treatment. CCK8 reagent was then added to each well for 1-2 h at 37 ℃. The OD value was measured at 450 nm in a microplate reader epoch2 (Bio-Tek, USA).

**EdU incorporation assay**

The EdU incorporation assay was performed in 24-well plates using an EdU incorporation assay kit (RiboBio Co., Ltd., C10310-3, China). Briefly, cells were cultured in a 24-well plate with indicated treatment for 24 h. Cells were labelled with 10 μM EdU for another 24 h at 37 °C. Cells were then fixed with 4% paraformaldehyde in PBS and stained with reaction cocktail. DAPI was subsequently used for nuclear staining, followed by imaging with a Nikon STORM Super-Resolution Microscope (Nikon A1 R+, Nikon, Japan).

**Flow cytometric analysis**

The mitochondrial membrane potential (MMP) was determined with a JC-1 assay kit (Beyotime, C2006, China). In addition, reactive oxygen species (ROS) were analysed using an ROS assay kit (Beyotime, S0033S, China). These experiments were performed based on the corresponding manufacturer’s instructions. Stained cells (>10,000) were examined by using a FACS flow cytometer (Beckman CytoFLEX, Beckman Coulter Life Sciences, USA). Flow Jo software was used to analyze the experimental data.

**Immunoblotting and immunoprecipitation**

Whole cell lysates were harvested and lysed in Radio-Immune Precipitation Assay (RIPA) lysis buffer and subjected to SDS-PAGE. Then, the proteins were transferred to PVDF membranes. After blocking with 5% nonfat milk blocking buffer for one hour at room temperature, the target proteins were detected by specific antibodies, for 12 h at 4°C. The PVDF membrane was then washed and probed with horseradish peroxidase-conjugated secondary antibodies for two hours at room temperature. The PVDF membranes were washed again and then visualized by enhanced chemiluminescence (Millipore, USA).

**Lentiviral transduction**

CRL4^CUL4A/DDB1^-silenced OCCs were generated by lentiviral infection. HEK293T cells were co-transfected with lentiviral packaging plasmids, psPAX2 and pMD2.G, along with shCUL4A, shDDB1 (sequences shown in Supplementary Table 4) or the corresponding control shRNA pLKO.1 plasmid to produce lentivirus. Supernatant from each HEK293T culture was then collected at 24-72 h and filtered (0.45 μm filter). OCCs were subsequently transduced with lentiviral-containing supernatant and then selected with DMEM containing puromycin (2 μg/mL; Sigma-Aldrich, USA) for 48-72 h.

**Statistical analysis**

All statistical analyses and graphics were performed using GraphPad 8 software. One-way ANOVA or Student’s *t* test was used to analyze significant differences. All data are displayed as the mean ± SEM of at least three independent experiments. p < 0.05 was considered statistically significant.

**Reference**

1 Valente, A. J. *et al.* A simple ImageJ macro tool for analyzing mitochondrial network morphology in mammalian cell culture. *Acta Histochem*. **119**, 315-326, (2017).

Figure S1


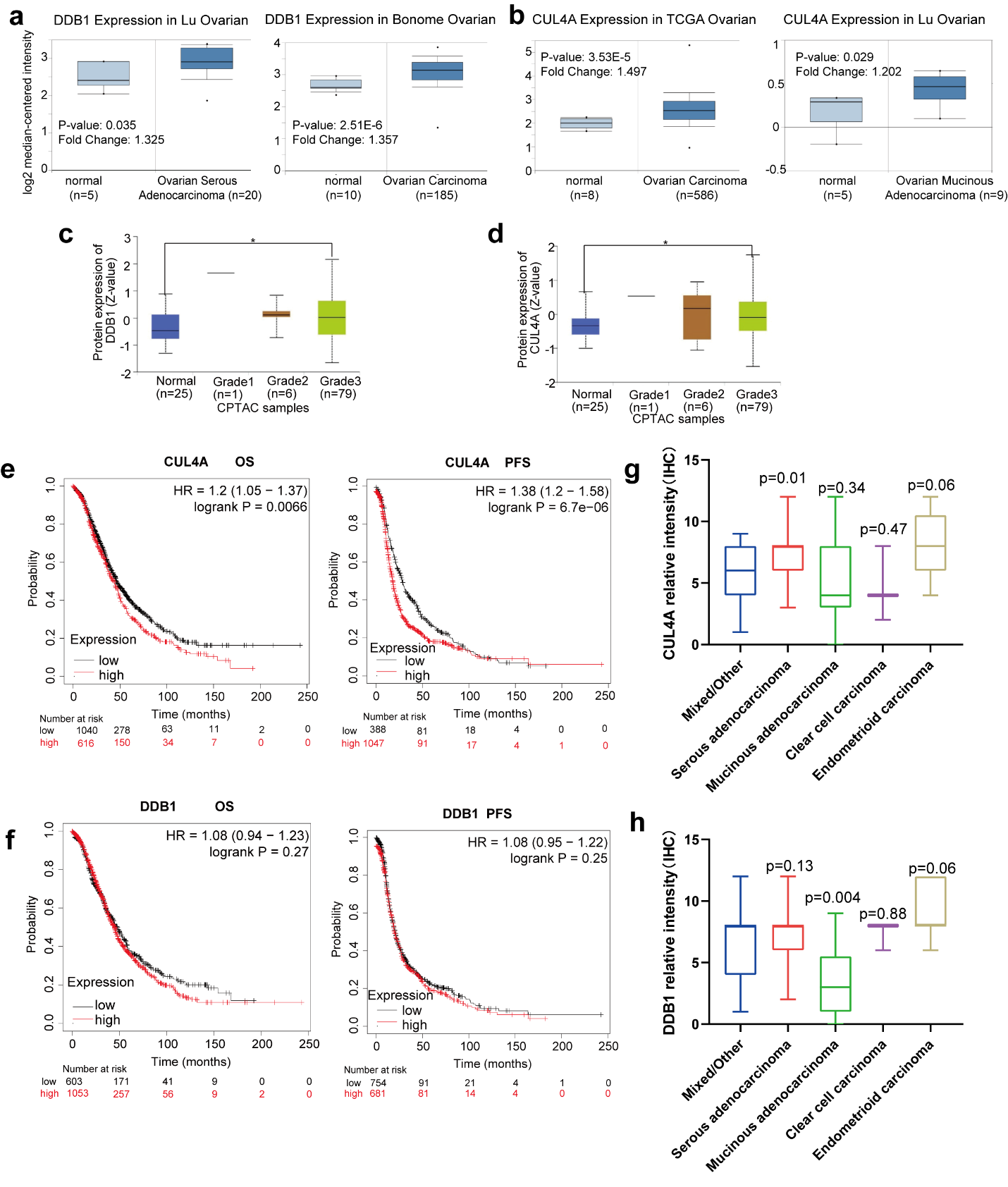


**Supplementary Figure. 1**

**CRL4^CUL4A/DDB1^ expression in OCCs is corelated with worse prognosis.**

**a.** Relative mRNA expression of DDB1 in the Lu ovarian and Bonome ovarian datasets.

**b.** Relative mRNA expression of CUL4A in the Lu ovarian and TCGA ovarian datasets.

**c-d. (c)** DDB1 and **(d)** CUL4A expression in various grade of ovarian cancer compared with normal ovarian tissues based on the CPTAC dataset. Raw data were obtained from the OncoLnc database.

**e-f.** Kaplan–Meier plot analysis with data from the OC RNA-seq project, showing the correlation of (e) CUL4A and (f) DDB1 expression with patient overall survival (OS) and progression free survival (PFS).

**g-h.** Mann-Whitney Test comparing (g) CUL4A and (h) DDB1 IHC staining scores of tissue microarray samples classified in each OC histotype to that of the samples classified with mixed histotypes, p<0.05 represents significant difference from mix histotype.

Figure S2


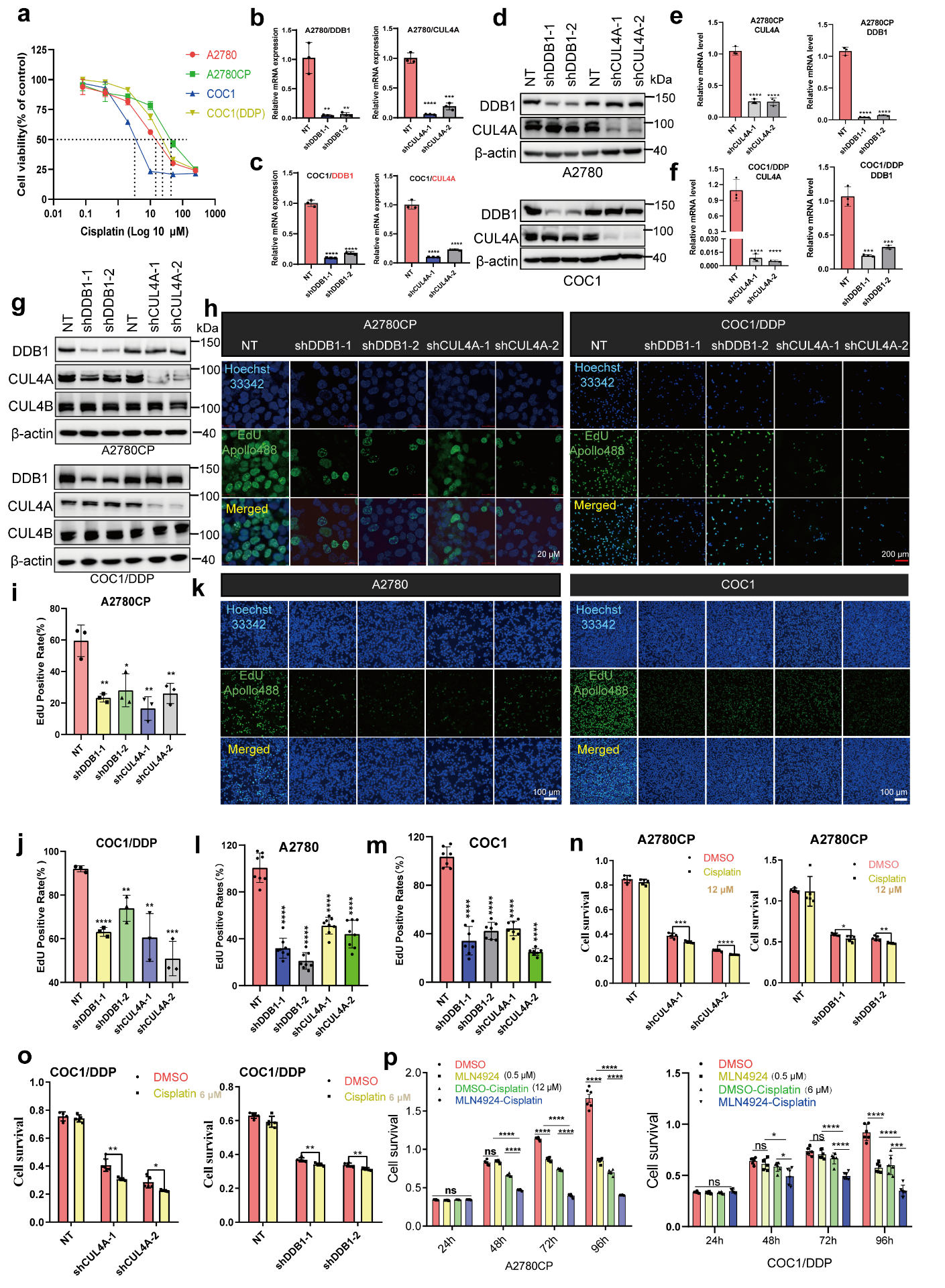


**Supplementary Figure. 2**

**CRL4^CUL4A/DDB1^ expression in OCCs is crucial for cisplatin resistance.**

**a.** Cell viability of COC1/DDP, COC1, A2780CP and A2780 cells detected by CCK8 assay after treated with cisplatin for 24 h, and the median inhibitory concentration (IC50) of cisplatin was indicated by the dash lines.

**b-c.** Relative DDB1 and CUL4A mRNA levels in (b) A2780 and (c) COC1 cell lines with or without shCUL4A/DDB1 knockdown. Data represent mean ± SEM normalized to 18S.

**d.** DDB1 and CUL4A protein expression in (upper panel) A2780 and (lower panel) COC1 cell lines with or without shCUL4A/DDB1 knockdown.

**e-f.** Relative DDB1 and CUL4A mRNA levels in (e) A2780CP and (f) COC1/DDP cell lines with or without shCUL4A/DDB1 knockdown. Data represent mean ± SEM normalized to 18S.

**g.** DDB1, CUL4B and CUL4A protein expression in (upper panel) A2780CP and (lower panel) COC1/DDP cell lines with or without shCUL4A/DDB1 kncokdown.

**h.** Cell proliferation of (left panel) A2780CP and (right panel) COC1/DDP cell lines detected by EdU incorporation assay. Representative images were shown.

**i-j.** Quantification of positive EdU staining signals in (i) A2780CP and (j) COC1/DDP cells, respectively. Data represent mean ± SEM from three replicates, ** *p*< 0.01, *** *p*< 0.001, **** *p*< 0.0001.

**k.** Cell proliferation of (left panel) A2780 and (right panel) COC1 detected by EdU incorporation assay. Scale Bar: 20 μm. Representative images were shown.

**l-m.** Quantification of positive EdU staining signals in (l) A2780 and (m) COC1, respectively. Data represent mean ± SEM from three replicates, ** *p*< 0.01, *** *p*< 0.001, **** *p*< 0.0001.

**n-o.** Representative proliferation of (n) A2780CP and (o) COC1/DDP cells with or without shCUL4A/DDB1 knockdown in the presence or absence of cisplatin for 24 h analyzed by CCK8 assay. Data represent mean ± SEM from three replicates.

**p.** Cell survival of (left panel) A2780CP and (right panel) COC1/DDP cells treated with MLN4924 (0.5 μM) followed by cisplatin treatment for the indicated time.

Figure S3

**
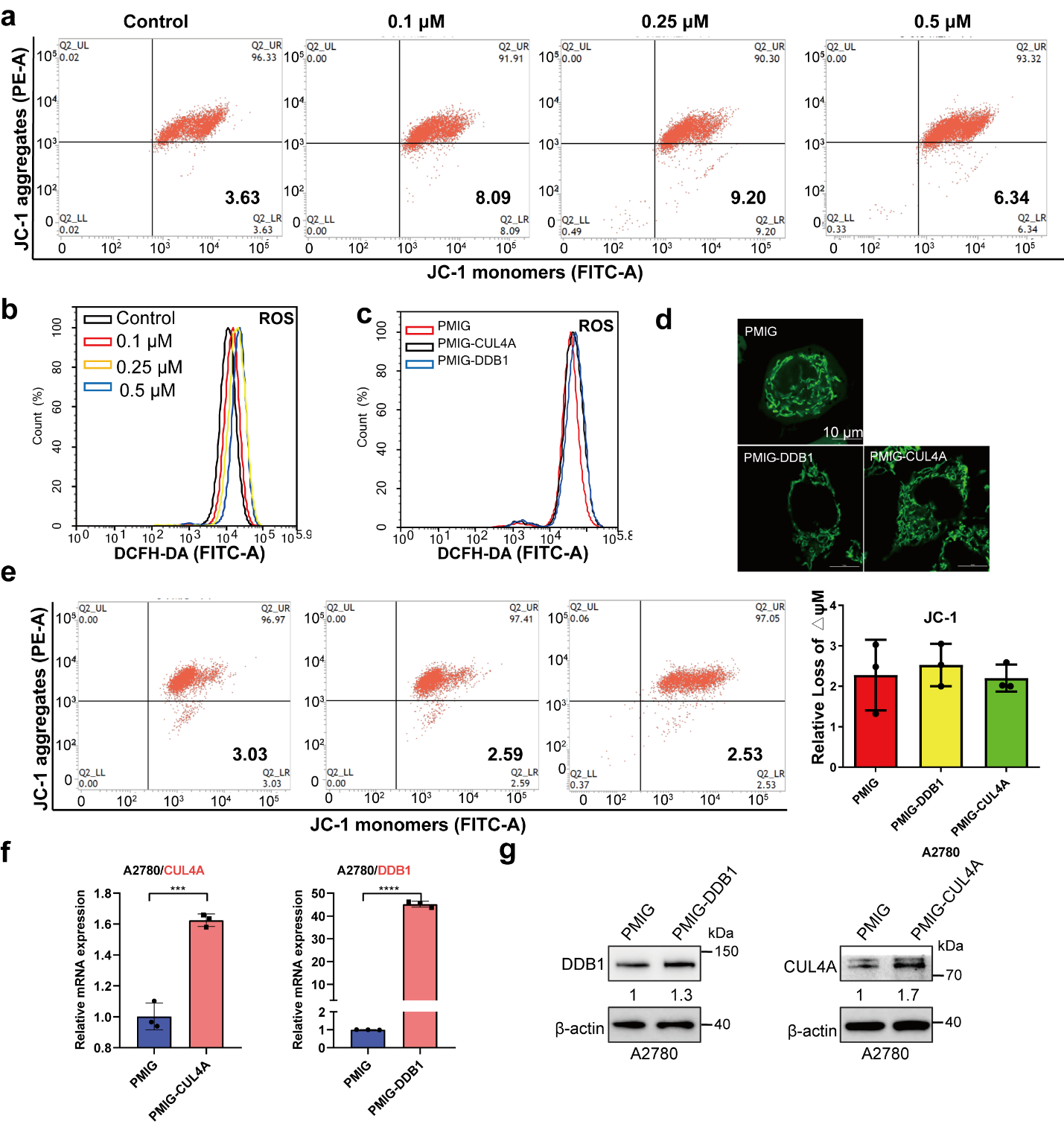
Supplementary Figure. 3**

**Overexpression of CRL4 had little effect on mitochondrial function of ovarian cancer.**

**a.** Mitochondrial membrane potential stained with JC-1 probe in A2780CP treated with indicated concentrations of MLN4924. Normal mitochondrial membrane potential (MMP) is shown in JC-1 dimers (JC-1 aggregates) and depolarized membrane potential is shown in JC-1 monomers.

**b.** ROS accumulation levels stained with DCFH-DA probe in A2780CP cells treated as in (A).

**c.** ROS accumulation levels stained with DCFH-DA probe in cisplatin-sensitive A2780 cells overexpressing CRL4^CUL4A/DDB1^.

**d.** Representative images of mitochondrial morphology in cisplatin-sensitive A2780 cells with CRL4^CUL4A/DDB1^ overexpression were stained with Mito Tracker Green. The images were captured by laser confocal microscope.

**e.** Mitochondrial membrane potential stained with JC-1 probe in Cisplatin-sensitive A2780 cells overexpressing CRL4^CUL4A/DDB1^. Normal mitochondrial membrane potential (MMP) is shown in JC-1 dimers (JC-1 aggregates) and depolarized membrane potential is shown in JC-1 monomers. Results were averaged from three independent experiments, measured in quadruplicate. Significance of differences was calculated using Student’s *t* test (****p*<0.001).

**f.** Relative (left panel) CUL4A and (right panel) DDB1 mRNA levels in A2780 cells infected with virus containing Cul4A/DDB1 overexpression vector respectively, or empty PMIG vector as control. Data represent mean ± SEM normalized to β-actin.

**g.** Western blot detection of (left panel) CUL4A and (right panel) DDB1 in A2780 cells infected with virus containing CUL4A/DDB1 overexpression vector respectively, or empty PMIG vector as control. β-actin serves as loading control.

Figure S4

**
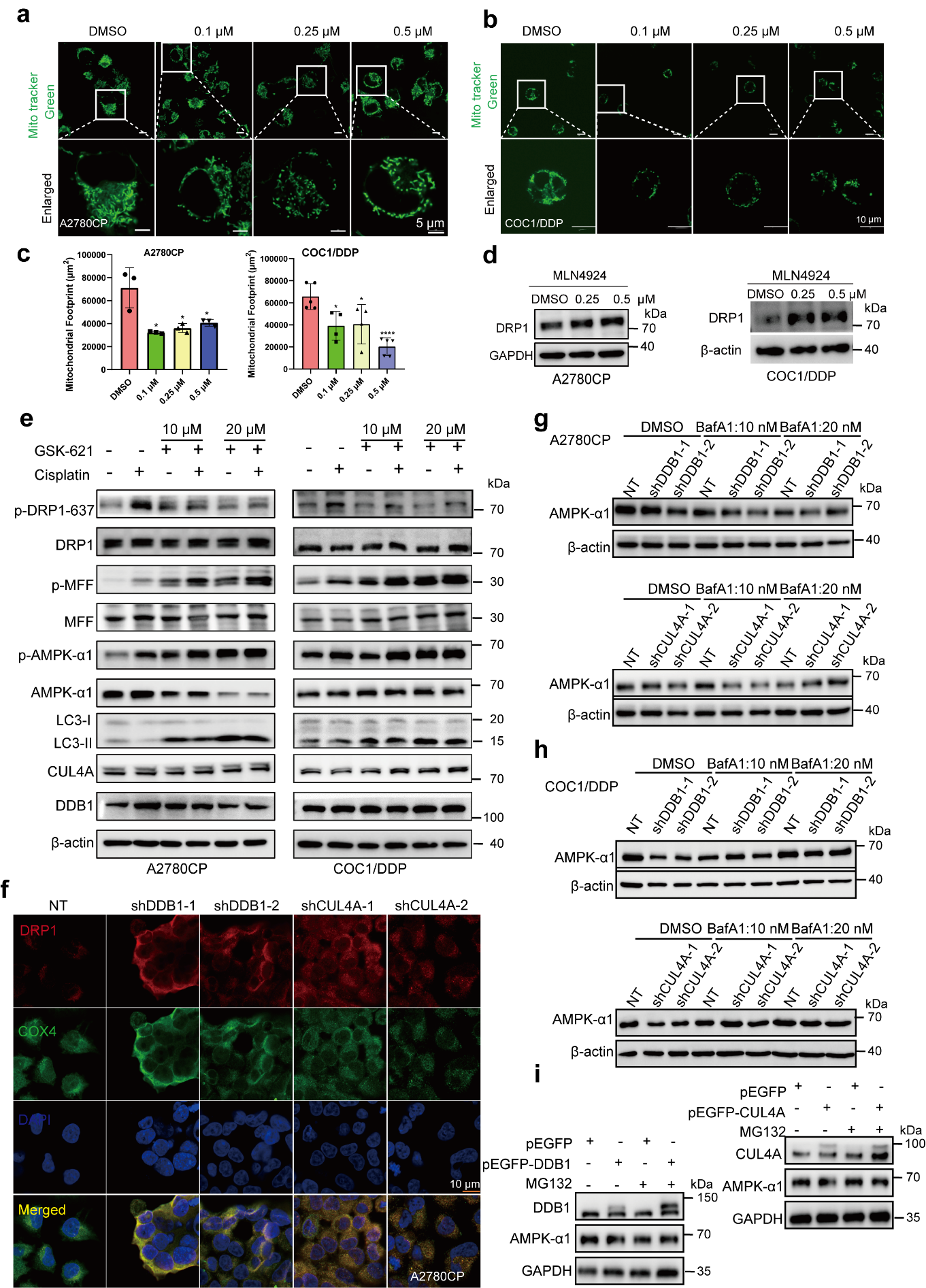
**

**Supplementary Figure. 4**

**Inhibition of CRL4^CUL4A/DDB1^ induced mitochondrial fragmentation along with mitochondrial translocation of Drp1 in cisplatin-resistant OCCs.**

**a-b.** Representative images of mitochondrial morphology stained with mitochondrial tracker Green in (a) A2780CP and (b) COC1/DDP treated with indicated concentrations of MLN4924.

**c.** Image analyses of the mitochondrial footprint in (left panel) A2780CP and (right panel) COC1/DDP treated with indicated concentrations of MLN4924. Significance of differences was calculated using Student’s *t* test (****p*<0.001).

**d.** DRP1 expression in A2780CP and COC1/DDP cells treated with indicated concentrations of MLN4924 was detected by western blot.

**e.** Immunoblotting of AMPK-α1, p-AMPK^Thr172^, MFF, p-MFF, DRP1, and p-DRP1^Ser637^ in (left panel) A2780CP and (right panel) COC1/DDP cells with or without GSK-621 treatment followed by cisplatin treatment for the indicated time.

**f.** Immunofluorescence analysis of the colocalization of endogenous DRP1 and COX4 in A2780CP cells with CRL4^CUL4A/DDB1^ knockdown. COX4 (Cytochrome c oxidase subunit 4 isoform 1) serves as a mitochondrial protein marker. Scale Bar:10 μm.

**g-h.** Immunoblot detection of AMPK-α1 in (g) A2780CP and (h) COC1/DDP CRL4-knockdown cells with indicated concentration of bafilomycin A1(BafA1) treatment. β-actin serves as a loading control.

**i.** Immunoblotting of AMPK-α1 in A2780CP cells overexpressing CRL4^CUL4A/DDB1^ with or without MG132 treatment.

Figure S5


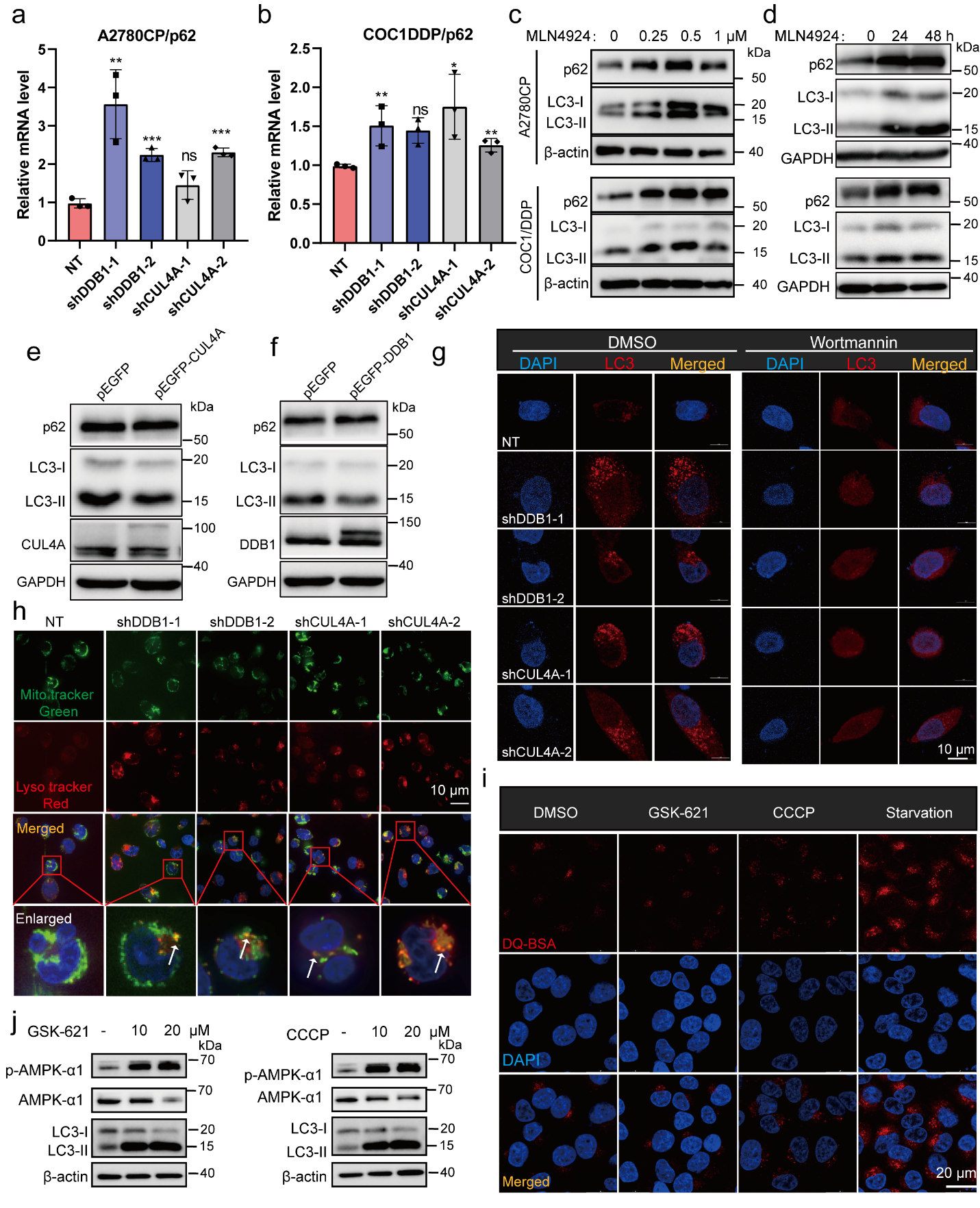


**Supplementary Figure. 5**

**Knockdown of CRL4 induced autophagy in cisplatin-resistant OCCs**

**a-b.** Relative mRNA levels of p62 in COC1/DDP and A2780CP cell lines with DDB1 or CUL4A knockdown. Data represent mean ± SEM normalized to 18S.

**c.** The expression of LC3B and p62 in OCCs treated with indicated concentrations of MLN4924 for 24 h.

**d.** The expression of LC3B and p62 in OCCs treated with 0.25 μM MLN4924 at for 24 or 48 h.

**e-f.** The expression of LC3B and p62 in A2780CP cells overexpressing (left panel) CUL4A or (right panel) DDB1.

**g.** Immunofluorescence analysis of LC3 in A2780CP cells after CRL4^CUL4A/DDB1^ knockdown with or without 10 µM of Wortmannin treatment for 24 h.

**h.** Colocalization of mitochondria with autolysosomes was analyzed by staining COC1/DDP cells with Lysotracker and Mito tracker. Red: Lysotracker red, green: Mito tracker green, orange-yellow: merge. Orange-yellow puncta were calculated as mitochondria having autolysosomes. White arrows show colocalization points. Scale bar :10 μm.

**i.** Representative images of A2780CP cells incubated with BODIPY-conjugated bovine serum (DQ-BSA, red) for 1 h followed by (second column) GSK621 and (third column) CCCP treatment for 24 h, or incubation with serum- and glucose-free medium (starvation). Scale bar: 20 μm.

**j.** Immunoblotting of AMPK-α1, p-AMPK^Thr172^, and LC3 in A2780CP followed by (left panel) GSK621 or (right panel) CCCP treatment for 24 h. β-actin serves as a loading control.

Figure S6

**
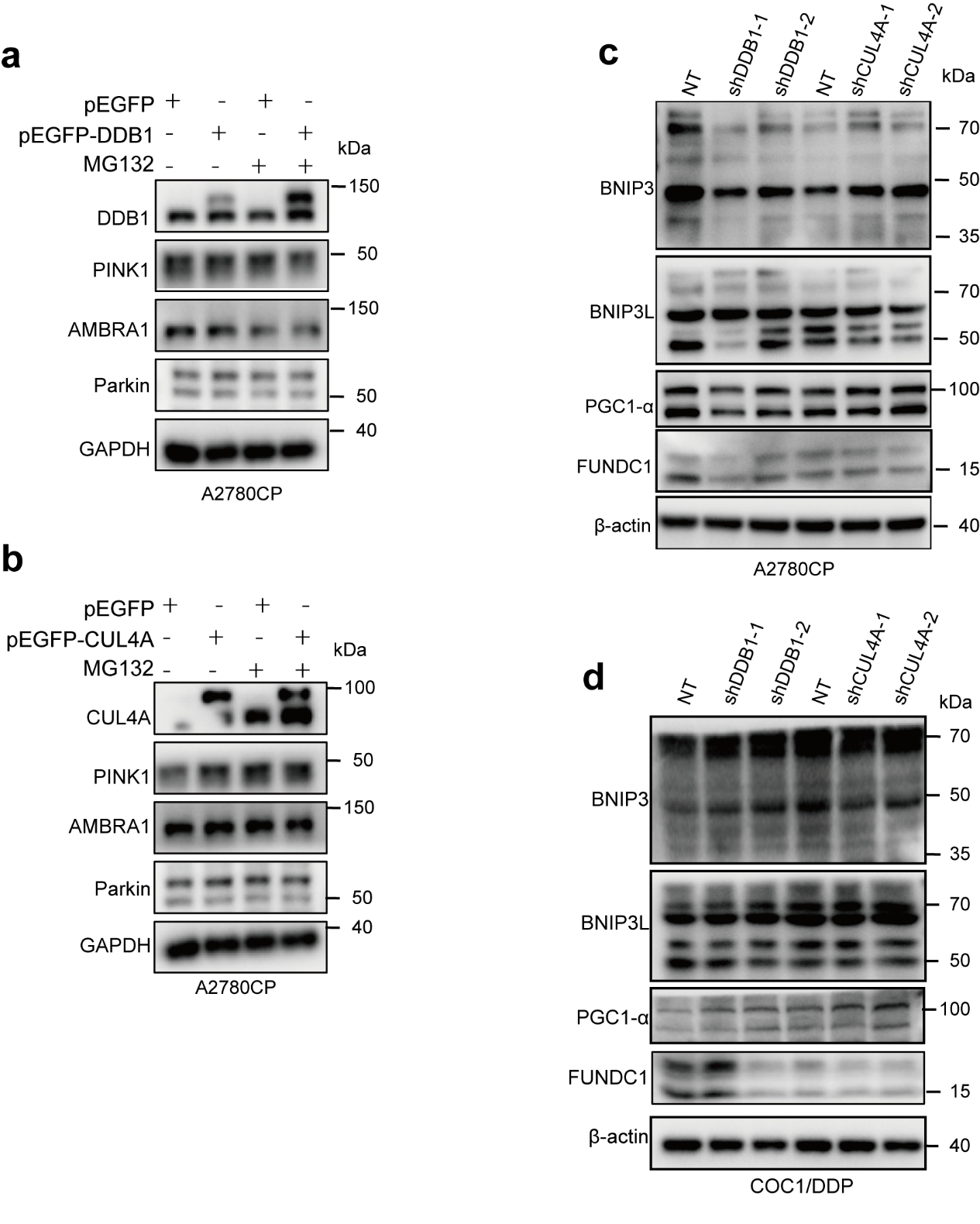
**

**Supplementary Figure. 6**

**Knocking down CRL4^CUL4A/DDB1^ stimulates mitophagy by inducing mitochondrial Parkin translocation in cisplatin-resistant OCCs.**

**a-b.** The expression of PINK1, Parkin and AMBRA1 in A2780CP cells overexpressing (left panel) DDB1 or (right panel) CUL4A with or without MG132 treatment.

**c-d.** The expression of BNIP3, BNIP3L, FUNDC1 and PGC1-α were shown in (left panel) A2780CP and (right panel) COC1/DDP cells with CRL4^CUL4A/DDB1^ knockdown. GAPDH serves as a loading control.
