# Supplementary material for: Targeting CRL4 suppresses chemoresistant ovarian cancer growth by inducing mitophagy
Source: Signal Transduct Target Ther. 2022 Dec 9;7:388. doi: 10.1038/s41392-022-01253-y (PMC9731993; doi:10.1038/s41392-022-01253-y)
Supplement: Supplementary file 2 — Supplementary Table S1 [file 41392_2022_1253_MOESM2_ESM.docx]

**Supplementary Table S1:** Patient information of tissue microarray for DDB1 staining.

| **Patients ID** | **Survival status** | **Overall Survival** | **Age** | **Histology type** | **Tumor stage** | **Recurrence(1,yes, 0,No)** | **Disease Free Survival** | **Response to platinum** |
| --- | --- | --- | --- | --- | --- | --- | --- | --- |
| J01A0867 | Dead | 40 | 71 | Mixed/Other | Ⅲ | 1 | 33 | Sensitive |
| J01A0868 | Live | 109 | 44 | serous adenocarcinoma | Ⅲ | 0 | 109 | Sensitive |
| D16A6602 | Dead | 21 | 47 | Mixed/Other | Ⅳ | 1 | 13 | Sensitive |
| J01A0869 | Live | 108 | 46 | serous adenocarcinoma | Ⅲ | 1 | 94 | Sensitive |
| J01A0871 | Live | 108 | 51 | serous adenocarcinoma | Ⅱ | 0 | 108 | Sensitive |
| J01A0872 | Dead | 52 | 64 | serous adenocarcinoma | Ⅲ | 1 | 38 | Sensitive |
| J01A0874 | Live | 107 | 36 | serous adenocarcinoma | Ⅱ | 0 | 107 | Sensitive |
| J01A0875 | Dead | 76 | 73 | serous adenocarcinoma | Ⅲ | 1 | 62 | Sensitive |
| J01A0876 | Live | 107 | 21 | Mixed/Other | Ⅱ | 0 | 107 | Sensitive |
| J01A0877 | Live | 107 | 40 | Mixed/Other | Ⅱ | 0 | 107 | Sensitive |
| J01A0878 | Dead | 81 | 62 | serous adenocarcinoma | Ⅲ | 1 | 71 | Sensitive |
| J01A0880 | Live | 106 | 58 | serous adenocarcinoma | Ⅲ | 1 | 44 | Sensitive |
| J01A0881 | Live | 106 | 46 | Endometrial carcinoma | Ⅱ | 0 | 106 | Sensitive |
| D99A0206 | Dead | 34 | 61 | Mixed/Other | Ⅳ | 1 | 22 | Sensitive |
| J01A0884 | Live | 105 | 55 | serous adenocarcinoma | Ⅲ | 1 | 58 | Sensitive |
| J01A0885 | Dead | 4 | 62 | serous adenocarcinoma | Ⅳ | 1 | 4 | Resistant |
| J01A0886 | Live | 103 | 40 | serous adenocarcinoma | Ⅲ | 1 | 91 | Sensitive |
| J01A0887 | Dead | 62 | 64 | Mixed/Other | Ⅲ | 1 | 4 | Resistant |
| J01A0888 | Live | 103 | 56 | mucinous adenocarcinoma | Ⅰ | 1 | 82 | Sensitive |
| J01A0889 | Live | 103 | 37 | serous adenocarcinoma | Ⅱ | 0 | 103 | Sensitive |
| J01A0890 | Dead | 18 | 73 | serous adenocarcinoma | Ⅲ | 1 | 15 | Sensitive |
| J01A0891 | Dead | 35 | 66 | serous adenocarcinoma | Ⅳ | 1 | 35 | Sensitive |
| J01A0892 | Dead | 44 | 62 | serous adenocarcinoma | Ⅲ | 1 | 34 | Sensitive |
| J01A0894 | Live | 99 | 58 | Mixed/Other | Ⅰ | 0 | 99 | Sensitive |
| J01A0896 | Dead | 4 | 49 | serous adenocarcinoma | Ⅳ | 1 | 3 | Resistant |
| J01A0898 | Live | 98 | 36 | Mixed/Other | Ⅲ | 1 | 51 | Sensitive |
| J01A0899 | Live | 98 | 52 | Mixed/Other | Ⅱ | 1 | 47 | Sensitive |
| J01A0902 | Live | 98 | 48 | serous adenocarcinoma | Ⅲ | 1 | 72 | Sensitive |
| J01A0903 | Live | 97 | 44 | Mucinous adenocarcinoma | **—** | —— | —— | Sensitive |
| J01A0904 | Live | 97 | 47 | Mixed/Other | Ⅲ | 1 | 79 | Sensitive |
| J01A0906 | Dead | 54 | 53 | serous adenocarcinoma | Ⅲ | 1 | 42 | Sensitive |
| J01A0907 | Live | 96 | 53 | Mixed/Other | Ⅲ | 0 | 96 | Sensitive |
| J01A0908 | Dead | 10 | 49 | serous adenocarcinoma | Ⅳ | 1 | 9 | Sensitive |
| J01A0909 | Dead | 32 |  | serous adenocarcinoma | Ⅲ | 1 | 13 | Sensitive |
| J01A0910 | Live | 96 | 63 | Mixed/Other | Ⅲ | 1 | 83 | Sensitive |
| D99A0207 | Dead | 64 | 73 | serous adenocarcinoma | Ⅳ | 1 | 27 | Sensitive |
| J01A0911 | Live | 95 | 65 | mucinous adenocarcinoma | Ⅲ | 1 | 11 | Sensitive |
| J01A0912 | Live | 95 | 51 | Mixed/Other | Ⅲ | 1 | 23 | Sensitive |
| J01A0914 | Dead | 8 | 57 | serous adenocarcinoma | Ⅲ | 1 | 3 | Resistant |
| J01A0916 | Dead | 27 | 46 | mucinous adenocarcinoma | Ⅳ | 1 | 18 | Sensitive |
| J01A0917 | Dead | 45 | 25 | serous adenocarcinoma | Ⅳ | 1 | 35 | Sensitive |
| J01A0918 | Live | 92 | 56 | Endometrial carcinoma | Ⅲ | 1 | 81 | Sensitive |
| J01A0919 | Live | 92 | 41 | serous adenocarcinoma | Ⅱ | 1 | 65 | Sensitive |
| J01A0920 | Dead | 37 | 47 | serous adenocarcinoma | Ⅳ | 1 | 21 | Sensitive |
| J01A0923 | Live | 90 | 60 | Mixed/Other | Ⅲ | 1 | 34 | Sensitive |
| J01A0924 | Live | 90 | 56 | mucinous adenocarcinoma | Ⅱ | 0 | 90 | Sensitive |
| J01A1013 | Live | 90 | 45 | mucinous adenocarcinoma | Ⅰ | 0 | 90 | Sensitive |
| J01A0928 | Live | 89 | 52 | Mixed/Other | Ⅱ | 0 | 89 | Sensitive |
| J01A0929 | Dead | 34 | 56 | serous adenocarcinoma | Ⅳ | 1 | 19 | Sensitive |
| J01A0932 | Dead | 4 | 60 | Clear cell carcinoma | Ⅲ | 1 | 1 | Resistant |
| J01A1014 | Live | 87 | 58 | Endometrial carcinoma | Ⅲ | 1 | 60 | Sensitive |
| J01A0933 | Dead | 20 | 56 | serous adenocarcinoma | Ⅳ | 1 | 11 | Sensitive |
| J01A0934 | Dead | 16 | 64 | serous adenocarcinoma | Ⅳ | 1 | 14 | Sensitive |
| J01A0936 | Live | 85 | 37 | Mixed/Other | Ⅰ | 0 | 85 | Sensitive |
| J01A0937 | Live | 84 | 55 | Clear cell carcinoma | Ⅲ | 1 | 28 | Sensitive |
| J01A0938 | Dead | 14 | 64 | serous adenocarcinoma | Ⅲ | 1 | 14 | Sensitive |
| K04A0063 | Dead | 75 | 54 | serous adenocarcinoma | Ⅳ | 1 | 65 | Sensitive |
| H02A0524 | Dead | 13 | 47 | Mixed/Other | Ⅳ | 1 | 8 | Sensitive |
| J01A0941 | Dead | 58 | 46 | Clear cell carcinoma | Ⅲ | 1 | 50 | Sensitive |
| J01A0942 | Live | 83 | 48 | serous adenocarcinoma | Ⅲ | 1 | 29 | Sensitive |
| J01A0944 | Dead | 40 | 66 | serous adenocarcinoma | Ⅳ | 1 | 40 | Sensitive |
| J01A0946 | Dead | 55 | 60 | serous adenocarcinoma | Ⅲ | 1 | 41 | Sensitive |
| J01A0948 | Live | 83 | 59 | Mixed/Other | Ⅲ | 1 | 20 | Sensitive |
| J01A0949 | Live | 83 | 70 | Mixed/Other | Ⅱ | 0 | 83 | Sensitive |
| J01A0950 | Live | 83 | 31 | Mixed/Other | Ⅱ | 0 | 83 | Sensitive |
| J01A0952 | Live | 82 | 37 | mucinous adenocarcinoma | Ⅲ | 1 | 30 | Sensitive |
| D16A6603 | Dead | 27 | 62 | Mixed/Other | Ⅳ | 1 | 14 | Sensitive |
| J01A0954 | Live | 82 | 53 | serous adenocarcinoma | Ⅱ | 1 | 27 | Sensitive |
| J01A0956 | Dead | 66 | 40 | Mixed/Other | Ⅲ | 1 | 40 | Sensitive |
| J01A0958 | Live | 82 | 40 | Mixed/Other | Ⅱ | 1 | 49 | Sensitive |
| J01A0959 | Dead | 34 | 60 | serous adenocarcinoma | Ⅲ | 1 | 18 | Sensitive |
| J01A0960 | Dead | 17 | 39 | Mixed/Other | Ⅱ | 1 | 10 | Sensitive |
| J01A0961 | Live | 81 | 45 | mucinous adenocarcinoma | Ⅲ | 1 | 51 | Sensitive |
| J01A0962 | Live | 81 | 58 | Mixed/Other | Ⅱ | 0 | 81 | Sensitive |
| J01A0964 | Dead | 18 | 44 | serous adenocarcinoma | Ⅲ | 1 | 10 | Sensitive |
| J01A0965 | Dead | 37 | 48 | serous adenocarcinoma | Ⅱ | 1 | 25 | Sensitive |
| J01A0967 | Live | 79 | 43 | serous adenocarcinoma | Ⅱ | 0 | 79 | Sensitive |
| J01A0968 | Dead | 41 | 37 | serous adenocarcinoma | Ⅲ | 1 | **23** | Sensitive |
| J01A0969 | Live | 79 | 42 | mucinous adenocarcinoma | Ⅱ | 0 | 79 | Sensitive |
| J01A0971 | Live | 79 | 48 | Mixed/Other | Ⅰ | 0 | 79 | Sensitive |
| J01A0973 | Dead | 27 | 41 | Mixed/Other | Ⅳ | 1 | 14 | Sensitive |
| J01A0974 | Dead | 12 | 40 | serous adenocarcinoma | Ⅲ | 1 | 10 | Sensitive |
| J01A0975 | Live | 78 | 25 | mucinous adenocarcinoma | Ⅲ | 1 | 71 | Sensitive |
| J01A0977 | Live | 78 | 42 | serous adenocarcinoma | Ⅱ | 1 | 39 | Sensitive |
| J01A0978 | Live | 77 | 47 | Mixed/Other | Ⅰ | 0 | 77 | Sensitive |
| J01A0979 | Live | 77 | 43 | Endometrial carcinoma | Ⅱ | 0 | 77 | Sensitive |
| J01A0980 | Live | 75 | 42 | mucinous adenocarcinoma | Ⅱ | 0 | 75 | Sensitive |
| J01A0981 | Dead | 20 | 45 | Mixed/Other | Ⅳ | 1 | 5 | Resistant |
| J01A0982 | Live | 75 | 53 | mucinous adenocarcinoma | Ⅱ | 0 | 75 | Sensitive |
| J01A0984 | Live | 72 | 59 | serous adenocarcinoma | Ⅱ | 1 | 18 | Sensitive |
| J01A0985 | Dead | 18 | 50 | serous adenocarcinoma | Ⅲ | 1 | 9 | Sensitive |
| J01A0986 | Dead | 7 | 57 | serous adenocarcinoma | Ⅳ | 1 | 7 | Sensitive |
| J01A0987 | Dead | 27 | 46 | serous adenocarcinoma | Ⅲ | 1 | 11 | Sensitive |
| J01A0988 | Dead | 32 | 67 | serous adenocarcinoma | Ⅳ | 1 | 28 | Sensitive |
| J01A0990 | Dead | 11 | 42 | Mixed/Other | Ⅳ | 1 | 8 | Sensitive |
| J01A0991 | Dead | 45 | 52 | serous adenocarcinoma | Ⅳ | 1 | 30 | Sensitive |
| J01A1015 | Live | 69 | 60 | Mixed/Other | Ⅲ | 1 | 63 | Sensitive |
| J01A0992 | Live | 69 | 68 | serous adenocarcinoma | Ⅱ | 1 | 5 | Resistant |
| J01A0993 | Dead | 39 | 56 | serous adenocarcinoma | Ⅲ | 1 | 33 | Sensitive |
| J01A0994 | Live | 68 | 52 | Endometrial carcinoma | Ⅱ | 1 | 26 | Sensitive |
| J01A0995 | Live | 68 | 54 | serous adenocarcinoma | Ⅲ | 1 | 41 | Sensitive |
| J01A0996 | Dead | 8 | 66 | serous adenocarcinoma | Ⅲ | 1 | 8 | Sensitive |
| J01A0997 | Dead | 29 | 46 | Endometrial carcinoma | Ⅲ | 1 | 29 | Sensitive |
| J01A0999 | Dead | 36 | 69 | Endometrial carcinoma | Ⅲ | 1 | 27 | Sensitive |
| J01A1000 | Dead | 25 | 47 | mucinous adenocarcinoma | Ⅲ | 1 | 2 | Resistant |
| J01A1016 | Dead | 29 | 66 | Endometrial carcinoma | Ⅲ | 1 | 23 | Sensitive |
| J01A1004 | Live | 65 | 44 | serous adenocarcinoma | Ⅰ | 0 | 65 | Sensitive |
| J01A1006 | Live | 64 | 66 | serous adenocarcinoma | Ⅱ | 1 | 42 | Sensitive |
| J01A1008 | Dead | 45 | 40 | serous adenocarcinoma | Ⅲ | 1 | 28 | Sensitive |
| J01A1009 | Live | 63 | 42 | Mixed/Other | Ⅰ | 0 | 63 | Sensitive |
| J01A1010 | Live | 63 | 41 | Mixed/Other | Ⅱ | 0 | 63 | Sensitive |
| J01A1011 | Dead | 51 | 62 | serous adenocarcinoma | Ⅱ | 1 | 46 | Sensitive |
| J01A1012 | Dead | 32 | 38 | Endometrial carcinoma | Ⅲ | 1 | 21 | Sensitive |
