# Supplementary material for: Targeting CRL4 suppresses chemoresistant ovarian cancer growth by inducing mitophagy
Source: Signal Transduct Target Ther. 2022 Dec 9;7:388. doi: 10.1038/s41392-022-01253-y (PMC9731993; doi:10.1038/s41392-022-01253-y)
Supplement: Supplementary file 4 — Supplementary Table S3 [file 41392_2022_1253_MOESM4_ESM.docx]

**Supplementary Table S3:** Primers used in the qPCR experiments

| Primer name | Sequence |
| --- | --- |
| DDB1-F  DDB1-R  CUL4A-F  CUL4A-R | 5′-CATTCCTCGCTCCATCCTGATG-3′  5′-CCTTCTTACGGTCGCTCAACAG-3′  5′-GAATGAGCGGTTCGTCAACCTG-3′  5′-CTGTGGCTTCTTTGTTGCCTGC-3′ |
| P62-F  P62-R  NDUFS6-F  NDUFS6-R  PDK1-F  PDK1-R  IDH2-F  IDH2-R  HMGCL-F  HMGCL-R  CYB5R3-F  CYB5R3-R  β-actin-F  β-actin-R  18S-F  18S-R | 5′-TGTGTAGCGTCTGCGAGGGAAA-3′  5′-AGTGTCCGTGTTTCACCTTCCG-3′  TGGAGACTCGGGTGATAGCGTG  GTGGTGCTGTCTGAACTGGAGC  CATGTCACGCTGGGTAATGAGG  CTCAACACGAGGTCTTGGTGCA  AGATGGCAGTGGTGTCAAGGAG  CTGGATGGCATACTGGAAGCAG  TGCTGTCATGCAGGAAGTGCCT  CTCCAAGTCCTGCCACAGAAGA  GGAAGATGTCTCAGTACCTGGAG  TTGTCAGGTCGGATGGCGAACT  5′- GTGAAGGTGACAGCAGTCGGTT-3′  5′- GAAGTGGGGTGGCTTTTAGGA-3′  5′- ACCCGTTGAACCCCATTCGTGA-3′  5′- GCCTCACTAAACCATCCAATCGG-3′ |
