# Supplementary material for: Targeting CRL4 suppresses chemoresistant ovarian cancer growth by inducing mitophagy
Source: Signal Transduct Target Ther. 2022 Dec 9;7:388. doi: 10.1038/s41392-022-01253-y (PMC9731993; doi:10.1038/s41392-022-01253-y)
Supplement: Supplementary file 5 — Supplementary Table S4 [file 41392_2022_1253_MOESM5_ESM.docx]

**Supplementary Table S4:** Oligonucleotides sequences for shRNA used in this study

| Primer name | sequence |
| --- | --- |
| shDDB1-1  shDDB1-2  shCUL4A-1  shCUL4A -2 | Forward oligo:  5’CCGGCCTTGATTGGTGTTGCCAGTTCTCGAGAACTGGCAACACCAATCAAGGTTTTTG-3’  Reverse oligo:  5’AATTCAAAAACCTTGATTGGTGTTGCCAGTTCTCGAGAACTGGCAACACCAATCAAGG-3’  Forward oligo:  5’CCGGCGACCGTAAGAAGGTGACTTTCTCGAGAAAGTCACCTTCTTACGGTCGTTTTTG-3’  Reverse oligo:  5’AATTCAAAAACGACCGTAAGAAGGTGACTTTCTCGAGAAAGTCACCTTCTTACGGTCG-3’  Forward oligo:  5’CCGGGTGTGGAGAAACAGCTATTAGCTCGAGCTAATAGCTGTTTCTCCACACTTTTTG-3’  Reverse oligo:  5’AATTCAAAAAGTGTGGAGAAACAGCTATTAGCTCGAGCTAATAGCTGTTTCTCCACAC-3’  Forward oligo:  5’CCGGGGACAAGAAGATGTTACTAAACTCGAGTTTAGTAACATCTTCTTGTCCTTTTTG-3’  Reverse oligo:  5’AATTCAAAAAGGACAAGAAGATGTTACTAAACTCGAGTTTAGTAACATCTTCTTGTCC-3’ |
